# Maternal smoking during pregnancy, other reproductive factors, and neonatal jaundice: A two-sample Mendelian randomization study

**DOI:** 10.18332/tid/220334

**Published:** 2026-06-24

**Authors:** Wenjing Geng, Donge Feng, Jingbin Qian

**Affiliations:** 1 Department of Neonatology, Neonatal Center, Beijing Children’s Hospital, Capital Medical University, National Center for Children’s Health, Beijing, China; 2 Pediatrics Department, Beijing Renhe Hospital, Beijing, China; 3 School of Medicine, Tsinghua University, Beijing, China

**Keywords:** Mendelian randomization, neonatal jaundice, reproductive factors, pregnancy diseases, causal associations

## Abstract

**INTRODUCTION:**

Neonatal jaundice is a common condition affecting 60% of term and 80% of preterm infants. This study aimed to explore the genetic prediction of associations between reproductive factors, including age at menarche, age at first birth, age at first intercourse, hypertension during pregnancy, gestational diabetes, and smoking during pregnancy, and neonatal jaundice using two-sample Mendelian randomization (MR).

**METHODS:**

A two-sample MR analysis was conducted utilizing genome-wide association studies (GWAS) summary statistics for six reproductive factors and neonatal jaundice from public databases, with data derived from individuals of European ancestry. The primary analysis employed the inverse variance weighted (IVW) method, while supplementary analyses included MR-Egger, weighted median, and weighted mode methods. Sensitivity analyses were assessed with Cochran's Q test, MR-Egger, MR-PRESSO test, and leave-one-out methods. Statistical power was calculated using the mRnd tool.

**RESULTS:**

Our findings indicated that genetically maternal smoking during pregnancy was associated with neonatal jaundice (OR=8.5227; 95% CI: 1.9246–37.7403, p=0.0048), with adequate statistical power (84.85%). No effect was observed for other reproductive factors on neonatal jaundice (All p>0.05), including age at menarche, age at first birth, age at first intercourse, hypertension during pregnancy, and gestational diabetes. Statistical power for these analyses was limited (<10%). In addition, sensitivity analyses confirmed these findings, indicating robustness against potential pleiotropy and heterogeneity.

**CONCLUSIONS:**

These results suggest that maternal smoking during pregnancy is associated with an increased risk of neonatal jaundice. Further research on the underlying biological mechanisms may help to better understand this association and its potential relevance for clinical practice.

## INTRODUCTION

Neonatal jaundice, characterized by the yellowing of the skin and sclera due to elevated serum bilirubin levels, is one of the most common clinical conditions encountered in newborns. It affects approximately 60% of term and 80% of preterm infants during the first week of life^1^. The clinical presentation of neonatal jaundice typically manifests within the first 2–4 days after birth and resolves within 1–2 weeks. However, the timing, progression, and severity can vary significantly among infants, necessitating close monitoring and timely intervention when required^2^. Understanding the risk factors associated with neonatal jaundice may contribute to preventive strategies and risk awareness prior to pregnancy.

Maternal reproductive factors have been increasingly recognized as potential contributors to the development of neonatal jaundice^3^. These factors encompass a range of physiological and lifestyle-related aspects of a mother’s reproductive history and pregnancy experience^4^. Research has suggested that advanced maternal age may be associated with an increased risk of neonatal jaundice, possibly due to higher rates of pregnancy complications and interventions^5,6^. In addition, pregnancy diseases also contributed to the development of neonatal jaundice. For instance, gestational diabetes has been linked to an elevated risk of neonatal hyperbilirubinemia, potentially mediated through increased fetal erythropoiesis and subsequent hemolysis^7,8^. Hypertensive disorders of pregnancy have also been associated with a higher incidence of neonatal jaundice, though the underlying mechanisms are not fully elucidated^9^. Finally, maternal smoking is also thought to be associated with neonatal jaundice, possibly driven by indirect mechanisms involving smoking-induced imbalances in the maternal–fetal hormonal milieu^10^. However, current understanding of the association between these factors and the disease remains incomplete, with observational studies often precluded from drawing definitive causal conclusions.

To address this gap, a Mendelian randomization (MR) approach was employed, which utilizes genetic variants as instrumental variables (IVs) to infer causality^11^. It can provide evidence for potential associations by leveraging genetic variants that are less prone to confounding and reverse causation, which is useful in informing public health policies and guiding interventions for various diseases and conditions^11,12^. This study aimed to investigate the possible relationships, if any, between maternal reproductive factors, including age at menarche, age at first birth, age at first intercourse, hypertension during pregnancy, gestational diabetes, and smoking during pregnancy, and neonatal jaundice through a two-sample MR study.

## METHODS

### Study design and data sources

**Figure 1:**
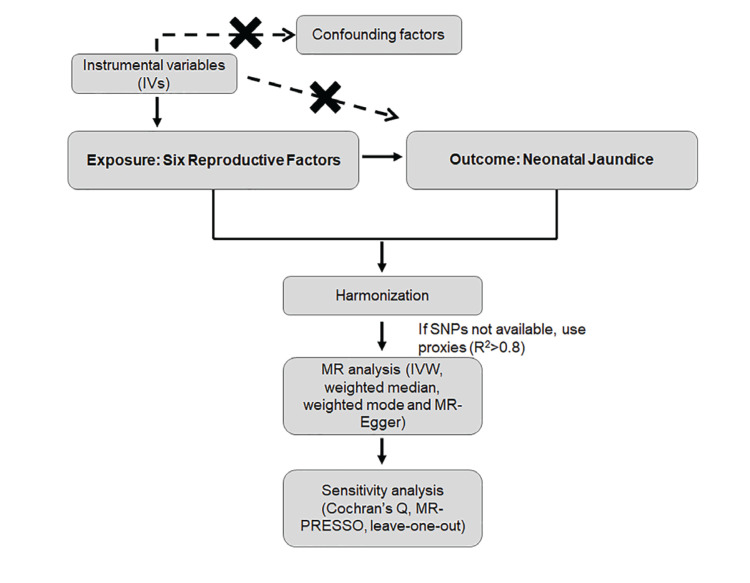
Flowchart of the Mendelian randomization (MR) study

A two-sample MR design was utilized to investigate relationships between six reproductive factors (age at menarche, age at first birth, age at first intercourse, hypertension during pregnancy, gestational diabetes, and smoking during pregnancy) and neonatal jaundice. The study adhered to the principles of the STROBE-MR checklist (Supplementary file), including the assumption that the genetic variants are associated with the exposure, independent of confounding factors, and only affect the outcome through the exposure^13^. A schematic representation of the MR workflow is provided (Figure 1). Detailed sample characteristics and GWAS information are provided in Supplementary file Table S1. A more detailed description of the methodology is given in the Supplementary file.

The GWAS summary statistics for neonatal jaundice were obtained from the FINN cohort, including 133 cases and 218608 controls. For reproductive factors, data were sourced from various studies and databases. For example, data on age at menarche were from a previous study, which involved up to 370000 women and 389 independent signals for age at menarche^14^. GWAS data on age at first birth and age at first sexual intercourse were from Day et al.^15^. GWAS data for gestational diabetes mellitus were obtained from Yang et al.^16^. All data used in this study were derived from public databases of European ancestry individuals. Detailed sample characteristics and GWAS information are provided in Supplementary file Table S1. All GWAS datasets used in this study were derived from individuals of European ancestry. Exposure data were obtained from the UK Biobank and previously published GWAS. Outcome data for neonatal jaundice were obtained from the FINN cohort. There was no sample overlap between the exposure and outcome GWAS datasets, as they were derived from independent sources. All data were accessed through established databases and did not require additional ethical approval.

### Instrumental variable selection

To ensure robustness, genetic variants that met predefined criteria were selected. First, SNPs significantly associated with gestational hypertension, gestational diabetes, and smoking during pregnancy were selected, meeting p<5×10^-6^ , due to the insufficient number of SNPs for subsequent analysis^17^; SNPs significantly associated with other exposures were selected^18^, meeting p<5×10^-8^. SNPs with a minor allele frequency (MAF) >0.01 were selected. Linkage disequilibrium (LD) effects between SNPs were eliminated based on the criteria of R^2^<0.001 and window size=10000kb^19^.

All exposure and outcome GWAS summary statistics were harmonized using the *harmonize_data()* function from the *TwoSampleMR* package in R. Palindromic SNPs with ambiguous allele frequencies (MAF >0.42) were excluded to avoid strand ambiguity, and effect alleles were forced to align consistently between exposure and outcome datasets to ensure correct directional estimation. No ambiguous SNPs remained after the harmonization process. Strand orientation was verified, and no strand issues were detected in the harmonized datasets.

When a selected IV was not present in the outcome summary data, a proxy SNP with high LD (R^2^ >0.8) to that IV was sought as a replacement using the *LDlinkR* package^20^. The F-value for each SNP in the IV was calculated to assess IV strength and exclude potential weak instrument bias between the IV and exposure factors. The calculation formula was: F=R^2^×(N-2)/(1-R^2^), where R^2^ is the proportion of exposure variance explained by the SNP in the IV. The requirement^19^ for the F-value is >10.

### MR analysis

An inverse variance weighted (IVW) method was employed as the primary analysis, complemented by weighted median, weighted mode, and MR-Egger regression^21^. MR methods estimate odds ratios (OR) and 95% confidence intervals (CIs) for the causal effect. The MR-Egger method was employed to investigate the presence of an intercept and provide precise causal effect estimates in the presence of pleiotropic bias^22^. In contrast, the weighted median approach relies on the assumption that at least 50% of the instrumental variables are valid for evaluating the causal relationship between exposure and outcome^23^. All analyses were performed using the TwoSampleMR package (V 0.5.11) within the R (V 4.0.5). Visual representations were generated through scatter plots and sensitivity analysis diagrams. To account for multiple testing due to the inclusion of four outcome factors, the false discovery rate (FDR) correction method was applied, with statistical significance defined^24^ as pFDR<0.05.

### Sensitivity analyses

Heterogeneity among IVs was assessed using Cochran’s Q test, with p>0.05 indicating no heterogeneity^25^. To account for the potential impact of genetic variation pleiotropy on association effect estimates, the MR-Egger regression approach was applied to investigate the presence of horizontal pleiotropy. A null intercept or lack of statistical significance in the MR-Egger regression suggests the absence of pleiotropy. Additionally, the MR-PRESSO method was employed to identify potential outlier single-nucleotide polymorphisms (SNPs) with p<0.05, and the causal association was reassessed following their exclusion^26^. This procedure corrects for horizontal pleiotropy. Leave-one-out analysis was conducted to remove each SNP step by step, calculate the meta-effect of the remaining SNPs, and observe whether the results change after removing each SNP^27^.

### Statistical power

Statistical power for each exposure–outcome pair was calculated using the *mRnd* online tool as recommended^28^. Power was estimated based on the two-sample Mendelian randomization design with a binary outcome.

## RESULTS

### Incorporation of instrumental variables

SNPs were extracted from the GWAS summary statistics of each phenotypic exposure under a well-recognized threshold. After screening, 526 SNPs were identified as related to reproductive factors as potential IVs. The mean value of the F-statistic of IVs is calculated to be >20, reflecting strong instrument validity (Supplementary file Table S2). In addition, in case of unavailability of exposure SNPs identified in the neonatal jaundice GWAS or vice versa, proxy-SNPs were used as recommended. There were 24 proxy SNPs used to replace SNPs when using ‘neonatal jaundice’ as the outcome (Supplementary file Table S3). This process ensured a robust set of instruments for MR analysis across different exposures.

### The effect of the reproductive factors on neonatal jaundice

**Figure 2:**
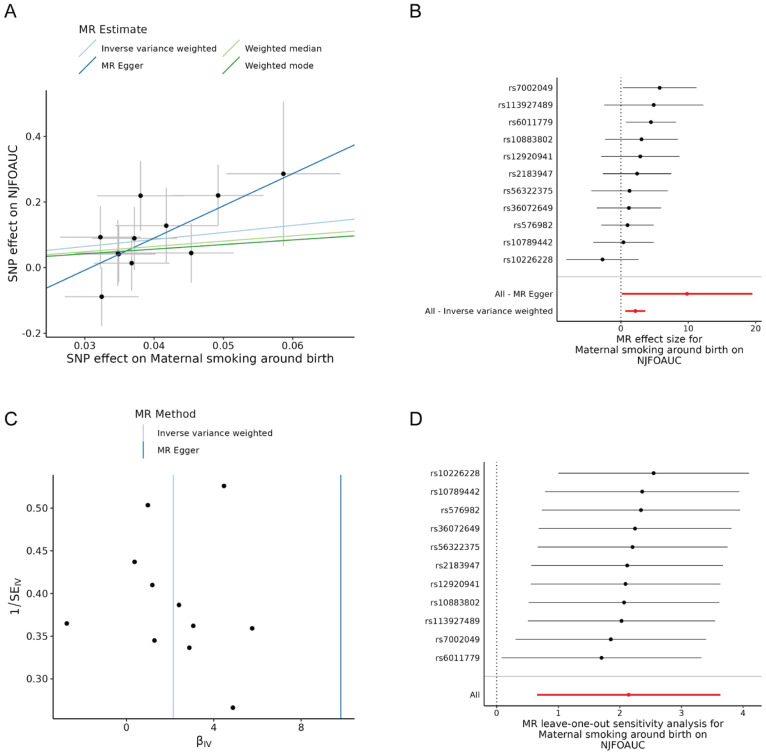
Analysis of the causal effect of maternal smoking around birth on neonatal jaundice through MR: A) A scatter plot illustrates the association between maternal smoking around birth and neonatal jaundice risk; B) The forest plot for the association between maternal smoking around birth and neonatal jaundice risk; C) A funnel plot for the maternal smoking around birth; D) Leave-one-out plot for the maternal smoking around birth

As shown in Table 1, the scatter plot (Figure 2A), and the forest plot (Figure 2B), the IVW analysis indicated a significant association between maternal smoking around birth (OR=8.52; 95% CI: 1.92–37.74, p=0.0048) and neonatal jaundice. No significant effects were detected for other reproductive factors (All p>0.05), such as age at first birth (OR=0.822; 95% CI: 0.572–1.181, p=0.2893). Meanwhile, supplementary analyses did not observe any significant association (Table 1).

**Table 1:** Two-sample MR analyses of causal relationships between reproductive factors and neonatal jaundice, exposure GWAS data from various sources, outcome GWAS data from FINN cohort (133 cases, 218608 controls, European ancestry)

*Exposure*	*Number of SNPs*	*Methods*	*OR (95% CI)*	*p*
**Age at first birth**	61	Inverse variance weighted	0.822 (0.5719–1.1813)	0.2893
		MR Egger	0.9968 (0.1768–5.6189)	0.9971
		Weighted median	0.9216 (0.5608–1.5144)	0.7474
		Weighted mode	1.0652 (0.3591–3.16)	0.9097
**Maternal smoking around birth**	11	Inverse variance weighted	8.5227 (1.9246–37.7403)	0.0048
		MR Egger	18359.4955 (1.122–300406611.5705)	0.0786
		Weighted median	5.0271 (0.637–39.6713)	0.1255
		Weighted mode	4.0758 (0.1648–100.8254)	0.4108
**Illnesses of the mother – high blood pressure**	8	Inverse variance weighted	1.0812 (0.918–1.2735)	0.3498
		MR Egger	1.4294 (0.9812–2.0823)	0.112
		Weighted median	1.1007 (0.9201–1.3168)	0.294
		Weighted mode	1.121 (0.8297–1.5146)	0.4811
**Diabetes or abnormal glucose tolerance complicating pregnancy**	6	Inverse variance weighted	0.9843 (0.8987–1.078)	0.7327
		MR Egger	0.9674 (0.7768–1.2046)	0.7816
		Weighted median	0.9995 (0.8876–1.1255)	0.9931
		Weighted mode	0.9958 (0.8575–1.1564)	0.9583
**Age at menarche**	197	Inverse variance weighted	0.906 (0.645–1.2722)	0.5687
		MR Egger	1.0473 (0.4011–2.7348)	0.9249
		Weighted median	1.0414 (0.5902–1.8377)	0.8886
		Weighted mode	1.1531 (0.3151–4.22)	0.8298
**Age at first sexual intercourse**	179	Inverse variance weighted	1.3323 (0.5218–3.402)	0.5486
		MR Egger	0.0936 (0.0014–6.1149)	0.2681
		Weighted median	2.4268 (0.6568–8.9672)	0.1837
		Weighted mode	7.2342 (0.2213–236.4496)	0.2675

Heterogeneity, directional pleiotropy, and horizontal pleiotropy among the genetic instruments were evaluated using MR-Egger regression analysis, Cochran’s Q test, and the MR-PRESSO test. The Q test results showed that there was no heterogeneity in our MR analysis (All p>0.05) (Table 2). MR-Egger regression analysis did not find pleiotropy (Table 2, Figure 2C). The MR-PRESSO test showed no horizontal pleiotropy among the tested SNPs (Global p>0.05, Supplementary file Table S4). The stability of our findings was validated by leave-one-out analyses, which verified that the exclusion of any individual SNP did not substantially affect the observed associations (Figure 2D).

**Table 2:** Results of the heterogeneity and horizontal pleiotropy tests for instrumental variables (two-sample MR design; based on the same datasets as in Table 1)

*Outcome*	*Exposure*	*Heterogeneity*		*Pleiotropy*	
		*Q statistic (IVW)*	*p*	*MR-Egger Intercept*	*p*
**Neonatal jaundice**	Age at first birth	75.052	0.091	-0.015	0.824
	Maternal smoking around birth	8.237	0.606	-0.303	0.151
	Illnesses of the mother – high blood pressure	12.337	0.09	-0.211	0.165
	Diabetes or abnormal glucose tolerance complicating pregnancy	5.022	0.413	0.023	0.87
	Age at menarche	208.552	0.256	-0.006	0.752
	Age at first sexual intercourse	204.509	0.084	0.043	0.203

### Statistical power

Statistical power calculations for each exposure-outcome pair were performed using the *mRnd* online tool. The analysis for maternal smoking during pregnancy achieved adequate power (84.85%), supporting the robustness of this finding (Supplementary file Table S5). For the other reproductive factors, the genetic instruments explained only a small proportion of variance (R^2^), resulting in limited statistical power (power <10%). Therefore, the absence of significant estimates for these exposures should not be interpreted as evidence of no causal effect, but rather as insufficient power to detect modest associations.

## DISCUSSION

This study provides evidence suggesting a link between maternal smoking during pregnancy and neonatal jaundice. In addition, there was no evidence from MR analyses indicating that other types of reproductive factors, increased or decreased the risk of neonatal jaundice. These results suggest that neonatal jaundice may represent an additional potential adverse outcome associated with maternal smoking during pregnancy.

Over the past decade, numerous studies have described the association between maternal smoking and increased risk of neonatal jaundice. A South African cohort study indicated that maternal smoking was not protective against neonatal jaundice^29^. However, different findings have been reported in the Asian cohort. For instance, a Chinese cohort found that maternal smoking was an independent risk factor for neonatal jaundice^30^. It is evident that previous studies have not reached a definitive conclusion regarding the causal relationship between maternal smoking during pregnancy and neonatal jaundice. This may be due to the influence of numerous confounding factors in these observational studies. This study provides further evidence at the genetic level in a European cohort, confirming a possible correlation between maternal smoking during pregnancy and neonatal jaundice across diverse ethnicities. However, these research discrepancies may stem from differences in ancestry/ethnicity, necessitating further investigation for verification. The observed association between maternal smoking and neonatal jaundice is biologically plausible. Cigarette smoke contains numerous toxic compounds, including polycyclic aromatic hydrocarbons and nicotine, which can cross the placental barrier and affect fetal development^31^. These toxins may interfere with the maturation of hepatic enzymes responsible for bilirubin metabolism, particularly UDP-glucuronosyltransferase 1A1 (UGT1A1)^32^, leading to impaired bilirubin conjugation and excretion in newborns^33^. Furthermore, maternal smoking has been associated with oxidative stress and inflammation, which may contribute to increased hemolysis and bilirubin production in the fetus^34^. The combination of increased bilirubin production and impaired clearance could explain the higher incidence of jaundice observed in infants born to mothers who smoked during pregnancy. Therefore, promoting smoking cessation during pregnancy is an important consideration for reducing the risk of neonatal jaundice. Hospitals and healthcare providers can effectively implement smoking cessation programs, promote smoke-free homes, vehicles, and public spaces, and consider smoking history and consider maternal smoking history as a predictor of neonatal jaundice. By addressing these risks, healthcare professionals can improve maternal and neonatal health outcomes and promote a healthier environment for pregnant women and their developing fetuses.

Moreover, previous observational studies have found that hypertension during pregnancy and gestational diabetes are associated with the incidence of neonatal jaundice^9,35^. However, in this study, no relationship was found between them. A critical concern with these observational studies is the variety and depth of confounding factors that can obscure the true relationship between reproductive diseases and neonatal jaundice. These studies often lack sufficient controls for a range of potential confounders, which include but are not limited to patient demographics such as age, hormone levels, and obesity^36^. Furthermore, these observational studies may also suffer from biases related to the methods by which data are collected. For example, the retrospective nature of data collection can lead to recall biases and selection biases, where individuals included in the study might not be representative of the general population^9^. In addition, this research indicates that there is no significant relationship between age at menarche, age at first birth, age at first sexual intercourse, and the incidence of neonatal jaundice. These findings suggesting that the timing of reproductive milestones in a woman’s life does not appear to influence the risk of neonatal jaundice in their offspring. Additionally, the physiological mechanisms governing the onset of menarche, first birth, and first sexual intercourse are complex and influenced by a myriad of genetic, environmental, and lifestyle factors that may not intersect with the etiological pathways leading to neonatal jaundice. More research is needed to confirm these conclusions.

### Strengths and limitations

This study presents several strengths. It represents a novel use of two-sample MR analysis to investigate potential relationships between the reproductive factors and neonatal jaundice. Traditional observational studies are more susceptible to bias due to confounding variables and the possibility of reverse causation. However, it is essential to consider the limitations of our study. For instance, because the participants in the datasets that we used were all of European ancestry, it remains to be seen through further research whether our study results can be generalized to other populations. Moreover, MR studies typically require a large sample size, and the current study sample size for neonatal jaundice (n=133) may be insufficient. Power calculations indicated that the analyses for several exposures were underpowered, and null findings should be interpreted with caution. Future research should replicate these findings in diverse populations and investigate potential biological mechanisms underlying this association.

## CONCLUSIONS

This study provides genetic evidence suggesting that maternal smoking during pregnancy is possibly associated with an increased risk of neonatal jaundice. These findings suggest that neonatal jaundice may represent an additional potential adverse outcome associated with maternal smoking during pregnancy.

## Supplementary Material



## Data Availability

The data supporting this research can be found in the Supplementary file.
