# Supplementary material for: Maternal smoking during pregnancy, other reproductive factors, and neonatal jaundice: A two-sample Mendelian randomization study
Source: Tob Induc Dis. 2026 Jun 24;24:10.18332/tid/220334. doi: 10.18332/tid/220334 (PMC13292640; doi:10.18332/tid/220334)
Supplement: Supplementary file 1 [file TID-24-95-s1.pdf]

STROBE-MR checklist of recommended items to address in reports of Mendelian randomization studies[1 2]

Item No.	Section		Checklist item	Page No.
1	**TITLE and ABSTRACT**		Indicate Mendelian randomization (MR) as the study's design in the title and/or the abstract if that is a main purpose of the study	1
	**INTRODUCTION**			
2	**Background**		Explain the scientific background and rationale for the reported study. What is the exposure? Is a potential causal relationship between exposure and outcome plausible? Justify why MR is a helpful method to address the study question	2
3	**Objectives**		State specific objectives clearly, including pre-specified causal hypotheses (if any). State that MR is a method that, under specific assumptions, intends to estimate causal effects	3
	**METHODS**			
4	**Study design and data sources**		Present key elements of the study design early in the article. Consider including a table listing sources of data for all phases of the study. For each data source contributing to the analysis, describe the following:	3
		a)	Setting: Describe the study design and the underlying population, if possible. Describe the setting, locations, and relevant dates, including periods of recruitment, exposure, follow-up, and data collection, when available.	3
		b)	Participants: Give the eligibility criteria, and the sources and methods of selection of participants. Report the sample size, and whether any power or sample size calculations were carried out prior to the main analysis	3
		c)	Describe measurement, quality control and selection of genetic variants	3
		d)	For each exposure, outcome, and other relevant variables, describe methods of assessment and diagnostic criteria for diseases	3
		e)	Provide details of ethics committee approval and participant informed consent, if relevant	
5	**Assumptions**		Explicitly state the three core IV assumptions for the main analysis (relevance, independence and exclusion restriction) as well assumptions for any additional or sensitivity analysis	3
6	**Statistical methods: main analysis**		Describe statistical methods and statistics used	4

		a)	Describe how quantitative variables were handled in the analyses (i.e., scale, units, model)	4
		b)	Describe how genetic variants were handled in the analyses and, if applicable, how their weights were selected	4
		c)	Describe the MR estimator (e.g. two-stage least squares, Wald ratio) and related statistics. Detail the included covariates and, in case of two-sample MR, whether the same covariate set was used for adjustment in the two samples	4
		d)	Explain how missing data were addressed	4
		e)	If applicable, indicate how multiple testing was addressed	/
7	**Assessment of assumptions**		Describe any methods or prior knowledge used to assess the assumptions or justify their validity	4
8	**Sensitivity analyses and additional analyses**		Describe any sensitivity analyses or additional analyses performed (e.g. comparison of effect estimates from different approaches, independent replication, bias analytic techniques, validation of instruments, simulations)	5
9	**Software and pre-registration**			5
		a)	Name statistical software and package(s), including version and settings used	
		b)	State whether the study protocol and details were pre-registered (as well as when and where)	-
	**RESULTS**			
10	**Descriptive data**			5
		a)	Report the numbers of individuals at each stage of included studies and reasons for exclusion. Consider use of a flow diagram	5
		b)	Report summary statistics for phenotypic exposure(s), outcome(s), and other relevant variables (e.g. means, SDs, proportions)	5
		c)	If the data sources include meta-analyses of previous studies, provide the assessments of heterogeneity across these studies	5
		d)	For two-sample MR:<br>i. Provide justification of the similarity of the genetic variant-exposure associations between the exposure and outcome samples<br>ii. Provide information on the number of individuals who overlap between the	5

			exposure and outcome studies	
11	**Main results**			6
		a)	Report the associations between genetic variant and exposure, and between genetic variant and outcome, preferably on an interpretable scale	6
		b)	Report MR estimates of the relationship between exposure and outcome, and the measures of uncertainty from the MR analysis, on an interpretable scale, such as odds ratio or relative risk per SD difference	6
		c)	If relevant, consider translating estimates of relative risk into absolute risk for a meaningful time period	6
		d)	Consider plots to visualize results (e.g. forest plot, scatterplot of associations between genetic variants and outcome versus between genetic variants and exposure)	/
12	**Assessment of assumptions**			6
		a)	Report the assessment of the validity of the assumptions	
		b)	Report any additional statistics (e.g., assessments of heterogeneity across genetic variants, such as I^2, Q statistic or E-value)	
13	**Sensitivity analyses and additional analyses**			6
		a)	Report any sensitivity analyses to assess the robustness of the main results to violations of the assumptions	6
		b)	Report results from other sensitivity analyses or additional analyses	6
		c)	Report any assessment of direction of causal relationship (e.g., bidirectional MR)	6
		d)	When relevant, report and compare with estimates from non-MR analyses	6
		e)	Consider additional plots to visualize results (e.g., leave-one-out analyses)	6
	**DISCUSSION**			
14	**Key results**		Summarize key results with reference to study objectives	6
15	**Limitations**		Discuss limitations of the study, taking into account the validity of the IV assumptions, other sources of potential bias, and imprecision. Discuss both direction and magnitude of any potential bias and any efforts to address them	7-8

16	**Interpretation**			6-8
		a)	Meaning: Give a cautious overall interpretation of results in the context of their limitations and in comparison with other studies	6-8
		b)	Mechanism: Discuss underlying biological mechanisms that could drive a potential causal relationship between the investigated exposure and the outcome, and whether the gene-environment equivalence assumption is reasonable. Use causal language carefully, clarifying that IV estimates may provide causal effects only under certain assumptions	6-8
		c)	Clinical relevance: Discuss whether the results have clinical or public policy relevance, and to what extent they inform effect sizes of possible interventions	6-8
17	**Generalizability**		Discuss the generalizability of the study results (a) to other populations, (b) across other exposure periods/timings, and (c) across other levels of exposure	6-8
	**OTHER INFORMATION**			
18	**Funding**		Describe sources of funding and the role of funders in the present study and, if applicable, sources of funding for the databases and original study or studies on which the present study is based	8
19	**Data and data sharing**		Provide the data used to perform all analyses or report where and how the data can be accessed, and reference these sources in the article. Provide the statistical code needed to reproduce the results in the article, or report whether the code is publicly accessible and if so, where	8
20	**Conflicts of Interest**		All authors should declare all potential conflicts of interest	8

This checklist is copyrighted by the Equator Network under the Creative Commons Attribution 3.0 Unported (CC BY 3.0) license.

1. Skrivankova VW, Richmond RC, Woolf BAR, Yarmolinsky J, Davies NM, Swanson SA, et al. Strengthening the Reporting of Observational Studies in Epidemiology using Mendelian Randomization (STROBE-MR) Statement. JAMA. 2021;under review.

2. Skrivankova VW, Richmond RC, Woolf BAR, Davies NM, Swanson SA, VanderWeele TJ, et al. Strengthening the Reporting of Observational Studies in Epidemiology using Mendelian Randomisation (STROBE-MR): Explanation and Elaboration. BMJ. 2021;375:n2233.

Table S2 The information of included IVs for exposure

chr.exposu	pos.exposu	beta.expos	se.exposur	pval.expos	SNP	effect_alle	other_alle	eaf.exposu	exposure
1	66635371	-0.0592	0.0105	1.87E-08	rs2069278	C	T	0.67	Age at first
1	1.54E+08	-0.0777	0.0108	7.01E-13	rs7269423	G	T	0.7087	Age at first
1	2.1E+08	0.0623	0.0105	3.37E-09	rs7516843	G	A	0.6352	Age at first
1	2.2E+08	0.0724	0.0125	6.16E-09	rs3767654	G	T	0.8459	Age at first
1	44338575	0.0677	0.0112	1.43E-09	rs2906457	C	A	0.3052	Age at first
1	91193049	0.0587	0.0101	6.68E-09	rs1092290	T	A	0.497	Age at first
1	22347396	0.0876	0.0151	6.86E-09	rs1240743	G	A	0.8449	Age at first
2	5891511	0.077	0.0121	2.14E-10	rs1188764	G	A	0.7366	Age at first
2	58068741	0.0606	0.0102	2.82E-09	rs1106090	A	G	0.3867	Age at first
2	1.01E+08	0.0627	0.0103	9.55E-10	rs7562372	C	T	0.3201	Age at first
2	1.04E+08	-0.071	0.0102	2.80E-12	rs1342578	G	A	0.4881	Age at first
2	1.66E+08	0.0703	0.0122	8.37E-09	rs1122825	G	A	0.7515	Age at first
2	12797853	-0.087	0.0158	3.76E-08	rs7277969	T	C	0.8887	Age at first
2	60475008	-0.0788	0.0103	1.58E-14	rs359240	A	G	0.4076	Age at first
2	2.15E+08	0.075	0.0132	1.20E-08	rs7398946	G	A	0.833	Age at first
2	51889019	-0.0741	0.0135	4.22E-08	rs1516198	G	A	0.1441	Age at first
2	1.56E+08	0.2459	0.0439	2.17E-08	rs8015328	A	C	0.98608	Age at first
3	47800216	-0.0667	0.0115	7.45E-09	rs1331920	A	T	0.7078	Age at first
3	83510683	0.0718	0.0125	9.46E-09	rs1376961	T	C	0.1998	Age at first
3	17953353	0.0616	0.0102	1.84E-09	rs9814726	A	T	0.5755	Age at first
3	49897830	-0.12	0.0101	7.52E-33	rs2681780	T	C	0.5099	Age at first
3	74883069	-0.0826	0.0133	4.75E-10	rs1191593	G	A	0.169	Age at first
4	3241845	-0.1263	0.0194	7.93E-11	rs362307	T	C	0.93738	Age at first
4	1.41E+08	0.0736	0.0103	7.79E-13	rs1731480	T	C	0.6521	Age at first
5	1.34E+08	0.0573	0.0102	1.90E-08	rs329122	A	G	0.5726	Age at first
5	45109961	0.0879	0.0134	4.51E-11	rs1215318	G	C	0.825	Age at first
5	60744339	-0.0561	0.0101	2.65E-08	rs1445979	A	T	0.6034	Age at first
6	32069806	-0.0824	0.0141	4.83E-09	rs3096695	C	G	0.1064	Age at first
6	34184938	0.0718	0.0131	4.49E-08	rs1080713	A	G	0.1958	Age at first
6	98527087	0.0672	0.0107	3.81E-10	rs9388090	T	C	0.5934	Age at first
6	1.45E+08	-0.087	0.0156	2.36E-08	rs1115536	A	C	0.8748	Age at first
6	1.52E+08	0.0858	0.0103	6.74E-17	rs2347867	A	G	0.3161	Age at first
7	2059761	0.0791	0.0129	9.46E-10	rs5598845	A	G	0.7873	Age at first
7	3445547	0.0704	0.0118	2.76E-09	rs2009182	C	T	0.2853	Age at first
7	1.14E+08	-0.0614	0.0103	2.09E-09	rs1859100	G	T	0.4682	Age at first
7	1.51E+08	0.0583	0.0102	9.87E-09	rs6964957	T	C	0.6252	Age at first
8	10815754	0.0623	0.0105	3.26E-09	rs1011195	G	C	0.4821	Age at first
8	1.46E+08	0.0661	0.0105	3.04E-10	rs1177421	T	C	0.502	Age at first
9	14745886	0.0651	0.0105	6.38E-10	rs7865801	A	G	0.3926	Age at first
9	23360417	0.069	0.0103	2.14E-11	rs1590949	G	C	0.5676	Age at first
10	1.07E+08	0.063	0.01	2.56E-10	rs1225038	G	A	0.3946	Age at first
10	1.19E+08	0.0956	0.0134	9.03E-13	rs6585429	G	A	0.1928	Age at first
11	30226528	0.0882	0.0141	3.77E-10	rs1103100	A	G	0.8588	Age at first

11	28668284	0.0575	0.0105	4.74E-08	rs7481939	G	A	0.6561	Age at first
12	1.23E+08	0.1079	0.0188	9.89E-09	rs1281547	T	C	0.92048	Age at first
12	56427808	0.0628	0.0107	3.90E-09	rs1702877	T	C	0.673	Age at first
12	84043145	-0.0801	0.011	3.81E-13	rs7958796	T	A	0.3489	Age at first
13	67143823	-0.1046	0.0178	4.37E-09	rs7870286	T	C	0.8986	Age at first
14	29604585	0.0898	0.0149	1.82E-09	rs176223	C	T	0.1322	Age at first
14	1.03E+08	-0.1001	0.0146	6.62E-12	rs7270471	G	T	0.8121	Age at first
14	27085685	-0.0677	0.0115	3.66E-09	rs1950402	G	A	0.2654	Age at first
14	72213258	0.0601	0.0109	3.24E-08	rs6574018	T	G	0.661	Age at first
15	99204101	-0.0538	0.0098	4.53E-08	rs8027457	C	T	0.4871	Age at first
15	74100132	0.0753	0.0135	2.56E-08	rs1452378	C	T	0.163	Age at first
16	59241949	-0.0749	0.0121	7.13E-10	rs1164674	T	G	0.2932	Age at first
16	90078724	0.0995	0.018	2.94E-08	rs7757604	T	C	0.94036	Age at first
17	43932797	-0.0888	0.0149	2.61E-09	rs1044536	C	G	0.7972	Age at first
17	56641200	0.0625	0.0102	1.01E-09	rs7359501	T	C	0.6213	Age at first
18	53260732	-0.0665	0.0103	1.06E-10	rs590076	A	G	0.6481	Age at first
18	35170045	0.0631	0.0109	7.82E-09	rs4799950	G	C	0.3469	Age at first
18	36510587	-0.0724	0.0127	1.21E-08	rs6022268	T	C	0.84	Age at first
19	13285293	0.0873	0.0119	2.59E-13	rs8110682	C	T	0.6342	Age at first
20	31097877	-0.071	0.0108	4.42E-11	rs293566	C	T	0.6859	Age at first
20	14840502	0.0549	0.01	3.55E-08	rs6079584	C	A	0.5815	Age at first
20	42001011	0.0699	0.0128	4.93E-08	rs6030812	A	G	0.1849	Age at first
21	46581798	-0.0573	0.0102	1.82E-08	rs394608	C	T	0.4553	Age at first
1	88829969	-0.0207	0.0035	1.60E-09	rs1156981	G	A	0.1143	Age at firs
1	91193049	0.0211	0.0021	2.10E-24	rs1092290	T	A	0.497	Age at firs
1	1.11E+08	-0.0129	0.0021	4.60E-10	rs2274568	A	G	0.4473	Age at firs
1	7522336	-0.0124	0.0021	4.20E-10	rs1962545	C	T	0.4751	Age at firs
1	44349405	0.0175	0.0025	6.80E-12	rs803679	A	G	0.2465	Age at firs
1	75001474	0.0145	0.0021	2.90E-12	rs7525548	T	A	0.4543	Age at firs
1	1.52E+08	-0.011	0.0021	3.90E-08	rs3507738	C	T	0.4851	Age at firs
1	2.05E+08	0.014	0.0024	2.20E-09	rs1124033	T	C	0.7366	Age at firs
1	2.33E+08	0.0134	0.0025	3.70E-08	rs1275777	A	G	0.8111	Age at firs
1	2.35E+08	-0.0122	0.0022	3.00E-08	rs6586405	A	C	0.6759	Age at firs
1	66481188	0.017	0.0021	6.20E-16	rs1392816	T	C	0.6243	Age at firs
1	78450517	-0.0195	0.0032	8.20E-10	rs3451743	A	C	0.90855	Age at firs
1	1.51E+08	0.0151	0.0025	1.90E-09	rs1204911	T	C	0.2117	Age at firs
1	1.56E+08	-0.0242	0.0042	8.10E-09	rs1476337	T	C	0.94732	Age at firs
1	96175101	-0.0117	0.0021	1.70E-08	rs1931263	T	G	0.5239	Age at firs
1	1.15E+08	0.0149	0.0027	2.30E-08	rs1085805	T	G	0.8121	Age at firs
2	60166832	-0.0221	0.0021	7.20E-27	rs6719762	C	T	0.5308	Age at firs
2	60463149	-0.0181	0.0021	4.10E-18	rs359271	C	T	0.4344	Age at firs
2	60777498	0.0123	0.0021	4.90E-09	rs6747099	C	G	0.5368	Age at firs
2	1.44E+08	0.0148	0.0021	1.90E-12	rs1320138	C	T	0.4254	Age at firs
2	22443840	-0.0118	0.0021	8.30E-09	rs4557006	A	G	0.5348	Age at firs
2	45062249	0.0318	0.0052	5.30E-10	rs6213419	T	C	0.95726	Age at firs

2	1.4E+08	0.0162	0.0022	3.90E-13	rs838042	A	G	0.3141	Age at firs
2	2.03E+08	0.0134	0.0024	2.90E-08	rs1339479	C	T	0.7644	Age at firs
2	2.13E+08	0.0126	0.0021	1.50E-09	rs714393	T	C	0.5249	Age at firs
2	622225	-0.0178	0.0027	1.10E-10	rs1320330	G	T	0.17	Age at firs
2	1.01E+08	0.0137	0.0021	3.80E-11	rs7566527	T	C	0.6133	Age at firs
2	1.74E+08	0.0164	0.0021	1.90E-15	rs7575189	A	G	0.4334	Age at firs
2	1.84E+08	0.0157	0.0025	4.10E-10	rs5630605	A	G	0.7922	Age at firs
2	6145158	0.0118	0.0021	3.10E-08	rs2091377	T	C	0.5646	Age at firs
2	44842145	-0.0191	0.0021	1.30E-19	rs6744794	G	C	0.3936	Age at firs
2	78018164	0.0224	0.004	1.40E-08	rs1168802	G	A	0.91551	Age at firs
2	1.04E+08	0.0165	0.0021	2.50E-15	rs1368546	C	T	0.4861	Age at firs
2	1.57E+08	0.0144	0.0021	2.30E-12	rs1226414	T	A	0.492	Age at firs
2	1.86E+08	-0.0307	0.0054	5.70E-09	rs1477251	T	C	0.97217	Age at firs
2	26948413	0.0141	0.0021	5.90E-12	rs1246372	A	G	0.5	Age at firs
2	32858637	0.0126	0.0021	2.20E-09	rs4952343	G	A	0.5547	Age at firs
2	1.62E+08	-0.012	0.0021	1.20E-08	rs1167898	A	G	0.5696	Age at firs
2	2.25E+08	0.0149	0.0022	1.10E-11	rs6748341	G	C	0.671	Age at firs
3	24908376	-0.0118	0.0021	1.60E-08	rs3460677	T	C	0.6014	Age at firs
3	35775115	0.0146	0.0021	1.30E-11	rs6772342	A	T	0.6193	Age at firs
3	1.18E+08	-0.015	0.0027	7.00E-09	rs705240	T	C	0.8201	Age at firs
3	50201924	-0.021	0.0021	2.10E-24	rs2188151	T	G	0.6034	Age at firs
3	84387950	-0.0217	0.0023	1.10E-20	rs1271459	C	A	0.7117	Age at firs
3	85650341	0.024	0.0022	1.20E-28	rs1125235	C	T	0.3698	Age at firs
3	54156598	-0.0183	0.0029	5.10E-11	rs1867234	G	A	0.8579	Age at firs
3	60870307	-0.0138	0.0025	1.20E-08	rs6776937	A	C	0.7594	Age at firs
3	88249922	-0.0242	0.0028	1.40E-17	rs1271470	G	A	0.164	Age at firs
3	1.08E+08	0.0206	0.0025	3.10E-17	rs7926940	A	G	0.7773	Age at firs
3	1.17E+08	-0.0172	0.0026	2.30E-11	rs4602427	G	C	0.1859	Age at firs
3	1.32E+08	-0.014	0.0021	8.90E-12	rs5639224	C	A	0.6441	Age at firs
3	3726156	-0.014	0.0021	1.80E-11	rs9809849	A	G	0.5895	Age at firs
3	17315758	0.014	0.0021	3.50E-11	rs2084572	G	A	0.5557	Age at firs
3	53773437	0.024	0.0028	1.20E-17	rs2612030	C	T	0.1431	Age at firs
3	70873278	0.0126	0.0021	1.90E-09	rs7618715	A	G	0.6014	Age at firs
4	25408838	0.0155	0.0024	1.30E-10	rs3481147	A	G	0.7833	Age at firs
4	62972597	0.0122	0.0022	3.50E-08	rs7671317	T	G	0.327	Age at firs
4	91590266	-0.0148	0.0024	6.60E-10	rs1051687	G	T	0.7565	Age at firs
4	60736871	0.0115	0.0021	1.60E-08	rs1251198	A	G	0.4672	Age at firs
4	28710551	0.0181	0.0028	2.80E-10	rs702	T	A	0.169	Age at firs
4	67825894	0.0157	0.0025	2.90E-10	rs993700	C	T	0.2326	Age at firs
4	1.13E+08	0.0222	0.0027	4.10E-16	rs1172908	A	G	0.8479	Age at firs
4	1.41E+08	0.0157	0.0021	1.00E-13	rs809955	A	G	0.661	Age at firs
5	31078814	0.0125	0.0023	4.40E-08	rs1317553	A	G	0.6928	Age at firs
5	60030791	-0.0136	0.0021	1.60E-10	rs7381195	A	T	0.3638	Age at firs
5	87847273	-0.0199	0.0021	2.00E-21	rs1265339	A	T	0.4493	Age at firs
5	45119647	0.0219	0.0027	1.10E-15	rs1252339	A	T	0.825	Age at firs

5	1.07E+08	0.0139	0.0022	4.30E-10	rs2406374	T	C	0.6799	Age at firs
5	1.55E+08	-0.0164	0.0024	4.20E-12	rs1317895	T	A	0.7445	Age at firs
5	30845465	-0.0128	0.0023	2.90E-08	rs7704530	A	G	0.2972	Age at firs
5	1.24E+08	0.0178	0.0029	4.50E-10	rs6077598	G	A	0.835	Age at firs
5	1.67E+08	-0.0138	0.0023	1.20E-09	rs1252309	C	T	0.7227	Age at firs
5	24921398	-0.017	0.0024	2.50E-12	rs435538	G	C	0.7744	Age at firs
5	46004640	-0.0149	0.0025	2.20E-09	rs1133382	C	T	0.7863	Age at firs
5	1.67E+08	0.0128	0.0021	8.20E-10	rs4868800	T	G	0.3926	Age at firs
6	67536056	-0.0163	0.0021	1.60E-14	rs7288733	C	T	0.6312	Age at firs
6	1E+08	0.0149	0.0022	1.70E-11	rs9403187	T	A	0.6362	Age at firs
6	1.52E+08	0.0275	0.0021	8.30E-38	rs1220471	T	C	0.3201	Age at firs
6	396321	0.014	0.0026	3.60E-08	rs1220359	T	C	0.8837	Age at firs
6	26319588	0.0135	0.0021	5.20E-10	rs766406	T	G	0.3539	Age at firs
6	50615935	0.0324	0.0038	1.40E-17	rs1415477	A	G	0.8867	Age at firs
6	1.64E+08	-0.0138	0.0024	1.00E-08	rs4709807	C	T	0.2306	Age at firs
6	23446691	-0.0171	0.0024	5.70E-13	rs767943	A	C	0.7127	Age at firs
6	52946320	0.017	0.0027	4.90E-10	rs222440	C	T	0.2038	Age at firs
6	87858691	0.0123	0.0021	7.30E-09	rs1925686	A	G	0.6282	Age at firs
6	1.25E+08	-0.0128	0.0021	4.90E-10	rs7452074	C	A	0.4334	Age at firs
7	1299334	0.0182	0.0033	1.90E-08	rs6966769	G	A	0.8598	Age at firs
7	32373639	0.0131	0.0022	2.30E-09	rs215639	T	C	0.3211	Age at firs
7	99119110	0.0226	0.0029	2.90E-15	rs7804551	G	A	0.837	Age at firs
7	1.14E+08	-0.0196	0.0021	1.40E-20	rs7783012	A	G	0.4761	Age at firs
7	3424686	0.0142	0.0022	9.50E-11	rs7785195	A	G	0.3429	Age at firs
7	21417556	-0.0155	0.0024	7.20E-11	rs6243969	A	G	0.7485	Age at firs
7	31330785	-0.0247	0.0034	2.50E-13	rs3585155	G	A	0.92644	Age at firs
7	1.4E+08	0.0156	0.0024	1.10E-10	rs1132471	C	T	0.6879	Age at firs
7	75147801	0.0142	0.0021	1.40E-11	rs794375	C	T	0.5537	Age at firs
7	1.05E+08	0.0218	0.0034	4.70E-11	rs1330722	A	G	0.1014	Age at firs
7	1966841	-0.0158	0.0021	2.70E-14	rs6978112	T	C	0.6044	Age at firs
7	1.33E+08	-0.0219	0.0029	2.10E-14	rs4728298	A	T	0.8231	Age at firs
7	1.22E+08	-0.0175	0.0028	1.60E-10	rs5893811	T	G	0.8201	Age at firs
8	26334103	0.0116	0.0021	3.40E-08	rs7008955	G	T	0.4771	Age at firs
8	51118559	-0.0155	0.0023	2.80E-11	rs7824756	C	T	0.7247	Age at firs
8	87680112	0.0179	0.0027	3.30E-11	rs7815125	A	T	0.1402	Age at firs
8	54396376	-0.0146	0.0026	1.30E-08	rs1585634	C	G	0.1879	Age at firs
8	73889570	-0.0123	0.0021	6.70E-10	rs1010452	C	T	0.4672	Age at firs
8	1.17E+08	-0.0134	0.0023	9.30E-09	rs1328059	G	C	0.2575	Age at firs
8	10706411	0.0182	0.0021	1.70E-17	rs1991651	G	C	0.3797	Age at firs
8	97825208	-0.0131	0.0021	2.50E-10	rs1095508	T	C	0.4761	Age at firs
8	42455166	0.0131	0.0021	2.70E-10	rs2974311	A	G	0.504	Age at firs
8	95489281	0.0137	0.0024	3.40E-08	rs7267482	C	T	0.7018	Age at firs
8	1.15E+08	-0.0135	0.0021	9.40E-11	rs9643087	T	C	0.4692	Age at firs
8	36842153	0.0123	0.0021	2.00E-08	rs1547351	A	T	0.5915	Age at firs
8	1.43E+08	0.0122	0.0021	8.90E-10	rs8180995	G	A	0.5378	Age at firs

9	86327243	0.0177	0.0029	3.70E-09	rs3447	G	C	0.8459	Age at firs
9	14529836	-0.0145	0.0024	7.90E-10	rs1237653	C	A	0.7396	Age at firs
9	23352293	0.0126	0.0021	1.20E-09	rs1255451	C	T	0.5736	Age at firs
9	81512820	0.0129	0.0022	5.40E-09	rs1074657	G	A	0.673	Age at firs
9	1.09E+08	-0.0151	0.0022	1.10E-11	rs2176337	T	A	0.673	Age at firs
9	96392182	0.0121	0.0022	1.70E-08	rs1099281	A	G	0.6352	Age at firs
9	16347927	0.013	0.0022	4.20E-09	rs4961705	C	G	0.6541	Age at firs
9	1.28E+08	0.0125	0.0022	1.40E-08	rs1912428	A	G	0.6918	Age at firs
9	1.35E+08	0.0144	0.0022	4.20E-11	rs4246175	A	T	0.3191	Age at firs
10	10922977	0.0139	0.0021	4.50E-11	rs2093623	A	G	0.5258	Age at firs
10	9973015	0.0125	0.0021	3.80E-09	rs7091634	G	A	0.6292	Age at firs
10	1.2E+08	0.0151	0.0028	2.70E-08	rs1128801	G	A	0.832	Age at firs
10	1.34E+08	-0.0165	0.0021	1.20E-15	rs7079070	A	G	0.5706	Age at firs
10	11205224	-0.0156	0.0028	2.90E-08	rs7909331	G	A	0.8459	Age at firs
10	1.19E+08	0.0175	0.0024	9.20E-13	rs1074923	C	G	0.2545	Age at firs
10	1.34E+08	0.0131	0.0024	1.70E-08	rs2130894	T	C	0.7008	Age at firs
10	63239803	-0.0166	0.0028	3.70E-09	rs2650705	G	A	0.1372	Age at firs
10	97941022	-0.0154	0.0022	5.70E-13	rs6185697	C	T	0.6928	Age at firs
10	1.05E+08	-0.0136	0.0022	5.80E-10	rs1224438	A	G	0.6372	Age at firs
10	1.06E+08	0.0198	0.0021	2.70E-21	rs3896224	G	A	0.5497	Age at firs
11	12875312	0.0148	0.0023	2.30E-10	rs1866710	G	A	0.2793	Age at firs
11	43771084	0.0151	0.0021	1.90E-13	rs3480422	G	A	0.6074	Age at firs
11	46342834	-0.014	0.0022	5.60E-10	rs7476	C	A	0.675	Age at firs
11	27734349	-0.0127	0.0022	1.10E-08	rs1229298	A	G	0.7058	Age at firs
11	79887549	0.0132	0.0021	1.10E-10	rs4439537	C	T	0.4523	Age at firs
11	1.13E+08	-0.0154	0.0021	5.00E-13	rs7110863	G	A	0.5666	Age at firs
11	28656064	0.0161	0.0022	6.60E-14	rs7942078	T	A	0.6561	Age at firs
11	1.06E+08	0.0135	0.0021	1.70E-10	rs590414	T	A	0.4911	Age at firs
11	1.27E+08	-0.0152	0.0021	4.90E-13	rs7927195	G	A	0.3718	Age at firs
12	23238326	0.0386	0.0058	5.20E-11	rs1126336	C	A	0.97018	Age at firs
12	41905017	0.016	0.0025	1.30E-10	rs1088008	A	G	0.8241	Age at firs
12	56468706	-0.0134	0.0022	8.40E-10	rs7955865	T	A	0.337	Age at firs
12	89745477	0.0119	0.0021	2.60E-08	rs2279574	A	C	0.4443	Age at firs
12	24195048	-0.0121	0.0021	4.50E-09	rs1995181	A	T	0.5457	Age at firs
12	84043146	-0.0149	0.0022	1.10E-11	rs7972441	A	C	0.3489	Age at firs
13	28104552	0.0199	0.003	1.50E-10	rs9581878	A	T	0.8449	Age at firs
13	55757388	0.0125	0.0022	4.20E-09	rs9536994	C	A	0.6571	Age at firs
13	69332015	-0.0128	0.0021	5.90E-10	rs2174752	T	G	0.5775	Age at firs
13	1.08E+08	-0.0114	0.0021	2.40E-08	rs9514600	G	C	0.494	Age at firs
13	60399045	-0.0159	0.0023	9.00E-13	rs341521	A	G	0.2972	Age at firs
13	59492828	-0.0159	0.0022	3.00E-13	rs9538248	A	C	0.669	Age at firs
14	41059928	-0.0202	0.0026	3.60E-14	rs1214746	A	G	0.837	Age at firs
14	58816212	0.0134	0.0021	5.30E-10	rs1287835	C	A	0.4751	Age at firs
14	1.03E+08	-0.0208	0.0026	4.00E-16	rs7151954	G	A	0.7833	Age at firs
14	93915929	-0.0135	0.0022	1.30E-09	rs4569188	A	G	0.3191	Age at firs

14	30726670	0.0353	0.0064	2.70E-08	rs7473773	T	A	0.98708	Age at firs
14	47367434	-0.0148	0.0021	1.50E-12	rs3007104	A	G	0.5885	Age at firs
14	94844947	0.0416	0.0074	1.20E-08	rs2892947	T	C	0.9831	Age at firs
14	98553482	-0.0156	0.0021	3.10E-13	rs7152323	G	A	0.5596	Age at firs
15	83240293	0.0148	0.0024	3.50E-10	rs783544	C	A	0.2406	Age at firs
15	91426560	0.0172	0.0021	1.10E-16	rs4702	A	G	0.4374	Age at firs
15	47684280	-0.0227	0.0025	2.10E-19	rs1290754	A	G	0.7932	Age at firs
15	97495941	-0.0138	0.0024	8.10E-09	rs7167444	T	G	0.7515	Age at firs
16	5825579	-0.0139	0.0023	3.80E-10	rs9923553	G	A	0.7207	Age at firs
16	75606878	0.0266	0.0045	5.50E-09	rs6564268	G	C	0.94732	Age at firs
16	735921	0.0163	0.0025	4.90E-11	rs763053	C	T	0.7326	Age at firs
16	12513797	0.0121	0.0023	2.70E-08	rs7201310	A	C	0.3012	Age at firs
16	24727064	-0.0146	0.0021	5.80E-12	rs7188873	G	A	0.3827	Age at firs
16	72505534	0.024	0.0031	4.90E-15	rs7651377	C	T	0.8897	Age at firs
16	90054704	-0.0168	0.0021	7.60E-16	rs1186642	G	C	0.34	Age at firs
16	49622284	-0.0179	0.003	2.10E-09	rs1244873	T	C	0.8658	Age at firs
17	47454507	-0.0165	0.0021	8.10E-15	rs2840636	T	C	0.6243	Age at firs
17	4938924	0.0113	0.0021	4.60E-08	rs410520	T	C	0.4851	Age at firs
17	65903326	-0.0149	0.0023	1.10E-10	rs6504551	G	T	0.7316	Age at firs
17	79095629	0.0129	0.0021	6.10E-10	rs7503604	A	C	0.5089	Age at firs
18	40231833	-0.0125	0.0021	3.40E-09	rs592278	A	G	0.5676	Age at firs
18	77579773	-0.0201	0.0025	3.90E-16	rs7236339	A	G	0.8161	Age at firs
18	44797697	0.0142	0.0021	1.80E-11	rs3415504	T	C	0.5596	Age at firs
18	50811573	0.0217	0.0038	1.50E-08	rs1046902	T	A	0.91252	Age at firs
18	53276589	-0.0153	0.0022	1.30E-12	rs1087158	T	G	0.6501	Age at firs
18	22647270	-0.0125	0.0021	4.60E-09	rs4800204	T	C	0.4135	Age at firs
19	4965064	-0.0123	0.0022	1.00E-08	rs1085398	A	G	0.6481	Age at firs
19	36252494	0.0122	0.0021	1.90E-09	rs807478	G	A	0.496	Age at firs
20	51510926	-0.0122	0.0022	3.30E-08	rs1609598	T	C	0.6859	Age at firs
20	62439274	0.0231	0.0039	2.50E-09	rs4809230	A	G	0.92744	Age at firs
20	14731057	-0.0356	0.0064	3.40E-08	rs1178311	T	C	0.98211	Age at firs
20	30864279	-0.0171	0.0028	1.80E-09	rs6058613	G	C	0.1312	Age at firs
21	40512129	-0.0275	0.005	3.20E-08	rs6517512	G	A	0.0388	Age at firs
1	7421139	0.029	0.005014	7.28E-09	rs845193	G	A	0.2515	Age at mer
1	8490320	-0.028	0.004663	1.91E-09	rs302719	G	T	0.6869	Age at mer
1	14138493	0.033	0.006022	4.26E-08	rs2744688	A	G	0.1402	Age at mer
1	21385436	-0.051	0.006796	6.18E-14	rs1212533	C	T	0.8459	Age at mer
1	41617914	0.044	0.0079	2.55E-08	rs2236129	G	C	0.92744	Age at mer
1	44029353	0.039	0.004777	3.26E-16	rs1121087	G	C	0.331	Age at mer
1	72733841	-0.034	0.004278	1.91E-15	rs1380995	T	A	0.5159	Age at mer
1	75002193	-0.048	0.004383	6.54E-28	rs1256698	A	G	0.4533	Age at mer
1	98375448	0.034	0.004692	4.27E-13	rs1116592	G	A	0.6918	Age at mer
1	1.03E+08	0.033	0.004379	4.86E-14	rs4908214	C	A	0.4553	Age at mer
1	1.51E+08	-0.082	0.014305	9.91E-09	rs1411643	T	C	0.96223	Age at mer
1	1.55E+08	0.026	0.004279	1.23E-09	rs9426832	G	A	0.5199	Age at mer

1	1.65E+08	0.08	0.006312	8.11E-37	rs3767357	G	A	0.8767	Age at mer
1	1.78E+08	0.069	0.005342	3.60E-38	rs506589	C	T	0.8131	Age at mer
1	2E+08	-0.031	0.00431	6.35E-13	rs1209136	T	C	0.504	Age at mer
1	2.06E+08	0.025	0.004321	7.24E-09	rs4951261	C	A	0.5974	Age at mer
2	466003	-0.11	0.01153	1.42E-21	rs6210418	A	G	0.95229	Age at mer
2	625029	0.071	0.005596	6.83E-37	rs7576624	T	C	0.17	Age at mer
2	25145173	0.029	0.004481	9.70E-11	rs5908689	A	T	0.5338	Age at mer
2	42984340	0.028	0.00512	4.52E-08	rs7605368	A	G	0.7545	Age at mer
2	56596394	-0.071	0.005656	3.80E-36	rs6704684	A	G	0.827	Age at mer
2	60456933	0.039	0.007152	4.96E-08	rs1188980	T	G	0.8847	Age at mer
2	61612865	-0.042	0.006196	1.21E-11	rs3463521	G	A	0.8549	Age at mer
2	69704941	0.033	0.005547	2.70E-09	rs2312205	G	A	0.8052	Age at mer
2	73536689	-0.11	0.013221	8.79E-17	rs5907224	T	C	0.97316	Age at mer
2	1.06E+08	0.047	0.004687	1.14E-23	rs2438086	G	A	0.4433	Age at mer
2	1.42E+08	-0.042	0.005961	1.85E-12	rs3593505	T	G	0.8598	Age at mer
2	1.54E+08	-0.041	0.0071	7.72E-09	rs1732864	G	T	0.8926	Age at mer
2	1.57E+08	-0.071	0.005724	2.51E-35	rs1420588	G	C	0.8469	Age at mer
2	1.65E+08	0.03	0.005051	2.87E-09	rs883208	A	C	0.7326	Age at mer
2	1.84E+08	0.04	0.005674	1.80E-12	rs1093107	T	C	0.2167	Age at mer
2	2E+08	0.053	0.00453	1.27E-31	rs1093183	T	C	0.6402	Age at mer
2	2E+08	0.032	0.005169	5.99E-10	rs6710368	C	T	0.7157	Age at mer
2	2.03E+08	0.047	0.006632	1.37E-12	rs6714523	A	G	0.8588	Age at mer
2	2.1E+08	-0.056	0.006933	6.62E-16	rs1356379	T	C	0.8986	Age at mer
3	18442437	0.029	0.004484	1.00E-10	rs9867904	C	G	0.6461	Age at mer
3	24206463	-0.1	0.012783	5.15E-15	rs7303599	C	T	0.9662	Age at mer
3	24715135	-0.042	0.004446	3.47E-21	rs1984870	T	G	0.504	Age at mer
3	49559485	-0.027	0.004574	3.57E-09	rs1306242	G	A	0.333	Age at mer
3	51333959	-0.15	0.016218	2.27E-20	rs1386257	C	G	0.9841	Age at mer
3	86910133	-0.045	0.004422	2.54E-24	rs6225727	T	C	0.3917	Age at mer
3	88224304	0.039	0.006879	1.43E-08	rs4858934	C	T	0.1064	Age at mer
3	1.15E+08	0.046	0.007349	3.87E-10	rs7644997	G	A	0.0915	Age at mer
3	1.18E+08	-0.053	0.004197	1.47E-36	rs1093442	C	T	0.4821	Age at mer
3	1.28E+08	-0.034	0.004867	2.83E-12	rs2461794	A	G	0.7366	Age at mer
3	1.33E+08	-0.033	0.005836	1.56E-08	rs1735103	C	T	0.8469	Age at mer
3	1.37E+08	-0.038	0.005728	3.26E-11	rs6675297	A	C	0.8419	Age at mer
3	1.57E+08	0.034	0.004399	1.08E-14	rs900399	G	A	0.6103	Age at mer
3	1.72E+08	0.025	0.004515	3.08E-08	rs582780	G	A	0.6213	Age at mer
3	1.86E+08	-0.043	0.004315	2.18E-23	rs2300922	T	C	0.5954	Age at mer
4	3267668	-0.028	0.004241	4.04E-11	rs2798224	A	G	0.4245	Age at mer
4	28746210	-0.038	0.004928	1.25E-14	rs4473643	C	T	0.2435	Age at mer
4	45182527	0.041	0.004353	4.59E-21	rs1093839	G	A	0.5795	Age at mer
4	95143122	-0.037	0.004322	1.12E-17	rs3113862	G	A	0.6143	Age at mer
4	1.05E+08	-0.054	0.00579	1.10E-20	rs3733632	G	A	0.834	Age at mer
4	1.06E+08	0.035	0.005992	5.17E-09	rs1703531	C	A	0.837	Age at mer
4	1.31E+08	-0.027	0.004889	3.34E-08	rs3111740	A	C	0.2813	Age at mer

4	1.33E+08	-0.038	0.005823	6.77E-11	rs6231679	A	C	0.827	Age at mer
4	1.53E+08	-0.026	0.004652	2.29E-08	rs1001117	A	G	0.659	Age at mer
4	1.77E+08	-0.025	0.004485	2.49E-08	rs1312003	T	C	0.671	Age at mer
5	43134752	0.034	0.004697	4.51E-13	rs1007685	A	G	0.3231	Age at mer
5	52909927	-0.029	0.004287	1.34E-11	rs813301	T	C	0.3549	Age at mer
5	64020316	-0.067	0.012028	2.54E-08	rs8017094	G	T	0.95129	Age at mer
5	1.11E+08	0.038	0.005129	1.27E-13	rs247520	C	T	0.7624	Age at mer
5	1.34E+08	-0.065	0.006091	1.38E-26	rs6237997	G	T	0.834	Age at mer
5	1.38E+08	0.036	0.005611	1.40E-10	rs2240330	T	C	0.7763	Age at mer
5	1.54E+08	-0.028	0.004257	4.78E-11	rs7719067	G	A	0.4414	Age at mer
5	1.57E+08	0.035	0.00574	1.08E-09	rs437836	C	T	0.162	Age at mer
5	1.67E+08	-0.037	0.006117	1.46E-09	rs9647570	G	T	0.8668	Age at mer
5	1.69E+08	0.037	0.005151	6.80E-13	rs6864818	C	T	0.2256	Age at mer
5	1.79E+08	-0.025	0.004537	3.58E-08	rs4701140	A	G	0.4761	Age at mer
5	1.81E+08	0.034	0.0052	6.20E-11	rs2770957	G	C	0.7793	Age at mer
6	28441634	-0.042	0.006441	7.02E-11	rs1266300	T	C	0.8718	Age at mer
6	41893323	0.037	0.004213	1.61E-18	rs9349203	A	G	0.4573	Age at mer
6	54720275	-0.035	0.00421	9.35E-17	rs9475046	C	G	0.5408	Age at mer
6	56862310	0.038	0.005205	2.85E-13	rs6901192	G	C	0.7565	Age at mer
6	76449917	-0.032	0.004374	2.56E-13	rs6241476	C	G	0.674	Age at mer
6	76928081	-0.026	0.004473	6.16E-09	rs1321933	T	C	0.3887	Age at mer
6	77713859	0.041	0.005316	1.23E-14	rs1414186	T	G	0.8131	Age at mer
6	1E+08	-0.061	0.006289	3.04E-22	rs6931884	T	C	0.8986	Age at mer
6	1.01E+08	0.034	0.004821	1.76E-12	rs9376867	A	G	0.6988	Age at mer
6	1.01E+08	0.028	0.004371	1.49E-10	rs240788	G	T	0.4205	Age at mer
6	1.05E+08	0.12	0.004471	1.20E-158	rs395962	G	T	0.3429	Age at mer
6	1.27E+08	0.04	0.004377	6.36E-20	rs4897178	G	T	0.5447	Age at mer
6	1.28E+08	0.029	0.00489	3.03E-09	rs1321857	C	T	0.6869	Age at mer
6	1.48E+08	-0.031	0.005107	1.28E-09	rs9497905	A	C	0.7913	Age at mer
6	1.52E+08	0.033	0.004547	3.93E-13	rs6933660	A	C	0.669	Age at mer
7	41470093	-0.072	0.006231	7.02E-31	rs1079866	G	C	0.8598	Age at mer
7	74138121	0.041	0.005572	1.87E-13	rs2267812	C	A	0.8062	Age at mer
7	75191602	-0.027	0.004435	1.14E-09	rs794361	A	T	0.5288	Age at mer
7	93312679	0.026	0.004402	3.49E-09	rs6968642	T	C	0.6123	Age at mer
7	94186064	-0.024	0.004346	3.34E-08	rs15671	C	A	0.4066	Age at mer
7	1.22E+08	-0.029	0.004445	6.85E-11	rs1023730	T	G	0.6233	Age at mer
7	1.33E+08	-0.028	0.004288	6.56E-11	rs2042067	C	T	0.5408	Age at mer
8	3766880	0.035	0.004815	3.64E-13	rs2688318	G	C	0.3032	Age at mer
8	4560227	-0.048	0.004287	4.23E-29	rs2724961	C	T	0.4334	Age at mer
8	4845681	0.028	0.004896	1.07E-08	rs3441542	C	T	0.7147	Age at mer
8	25280800	-0.029	0.004857	2.37E-09	rs6185	G	C	0.7575	Age at mer
8	53877882	0.05	0.006665	6.30E-14	rs1691837	C	T	0.8678	Age at mer
8	54024806	-0.038	0.00529	6.79E-13	rs1254942	T	G	0.7614	Age at mer
8	78097805	0.042	0.004482	7.16E-21	rs4735765	T	A	0.664	Age at mer
8	78854425	0.027	0.004481	1.69E-09	rs4739183	T	C	0.3827	Age at mer

8	87319950	0.041	0.005187	2.70E-15	rs7465046	T	C	0.7435	Age at mer
8	1.05E+08	0.026	0.004502	7.68E-09	rs7775124	G	T	0.3489	Age at mer
8	1.41E+08	-0.042	0.005688	1.54E-13	rs1469039	A	G	0.836	Age at mer
9	1676170	0.03	0.004473	1.99E-11	rs2783994	A	G	0.3191	Age at mer
9	7174673	0.037	0.004285	5.85E-18	rs913588	A	G	0.5249	Age at mer
9	10249081	-0.026	0.004677	2.72E-08	rs4741022	T	A	0.328	Age at mer
9	11808441	0.034	0.005187	5.56E-11	rs2046549	C	G	0.7624	Age at mer
9	73888674	-0.024	0.004199	1.09E-08	rs6560193	T	C	0.4871	Age at mer
9	76832928	0.036	0.005387	2.34E-11	rs1086935	C	T	0.164	Age at mer
9	83285190	0.032	0.004444	5.97E-13	rs7864983	A	G	0.6203	Age at mer
9	86712623	0.042	0.004449	3.75E-21	rs1074673	G	A	0.5507	Age at mer
9	96258838	0.027	0.004422	1.02E-09	rs1082114	T	C	0.6382	Age at mer
9	1.09E+08	0.1	0.004548	3.70E-107	rs1015659	T	A	0.674	Age at mer
9	1.14E+08	-0.1	0.00759	1.21E-39	rs7852169	G	C	0.92048	Age at mer
9	1.27E+08	-0.031	0.004303	5.86E-13	rs6478680	A	G	0.5189	Age at mer
10	1729026	-0.04	0.004458	2.88E-19	rs7896371	T	C	0.5586	Age at mer
10	2718972	-0.039	0.007027	2.86E-08	rs928593	C	T	0.1093	Age at mer
10	51056858	0.027	0.004708	9.73E-09	rs6184690	T	C	0.672	Age at mer
10	65191645	-0.025	0.004343	8.62E-09	rs7924036	T	G	0.492	Age at mer
10	74071178	0.026	0.004679	2.75E-08	rs4746113	A	G	0.67	Age at mer
10	97877320	0.042	0.004749	9.22E-19	rs1172955	A	T	0.332	Age at mer
10	1.05E+08	-0.025	0.004339	8.31E-09	rs1119154	G	A	0.5855	Age at mer
10	1.21E+08	0.028	0.004388	1.76E-10	rs1040013	A	G	0.4254	Age at mer
10	1.24E+08	0.05	0.007869	2.10E-10	rs7077302	G	C	0.0755	Age at mer
10	1.27E+08	-0.028	0.004253	4.57E-11	rs6597884	C	T	0.5795	Age at mer
11	237087	0.031	0.004956	3.96E-10	rs1045288	G	A	0.2684	Age at mer
11	8404501	-0.037	0.004385	3.23E-17	rs1693795	G	A	0.6064	Age at mer
11	13324530	0.048	0.004714	2.40E-24	rs1083202	A	G	0.2734	Age at mer
11	16786905	0.036	0.005857	7.94E-10	rs6137145	A	G	0.8519	Age at mer
11	27686196	-0.034	0.004565	9.52E-14	rs1076765	T	G	0.3032	Age at mer
11	29118542	0.032	0.005286	1.42E-09	rs7478970	A	G	0.8101	Age at mer
11	30317733	-0.035	0.005713	9.00E-10	rs1103104	G	T	0.839	Age at mer
11	43613426	-0.029	0.004465	8.27E-11	rs2625387	A	G	0.4036	Age at mer
11	46064974	-0.036	0.004766	4.26E-14	rs953230	A	G	0.3191	Age at mer
11	65473798	0.029	0.004862	2.45E-09	rs1075076	A	C	0.2903	Age at mer
11	78027488	-0.045	0.005865	1.68E-14	rs4945266	G	A	0.8231	Age at mer
11	84780098	-0.032	0.00517	6.05E-10	rs4402316	C	G	0.7167	Age at mer
11	94085099	0.044	0.007689	1.05E-08	rs1135575	T	C	0.90457	Age at mer
11	1.01E+08	-0.047	0.004462	6.04E-26	rs1089514	C	A	0.3628	Age at mer
11	1.23E+08	-0.06	0.004193	1.90E-46	rs7114175	T	A	0.506	Age at mer
12	2475403	0.024	0.0044	4.91E-08	rs1106220	C	T	0.5895	Age at mer
12	17126283	-0.12	0.01806	3.04E-11	rs7753042	G	A	0.97117	Age at mer
12	49399132	0.077	0.01282	1.90E-09	rs1126930	C	G	0.97614	Age at mer
12	50263148	0.041	0.004439	2.55E-20	rs7132908	A	G	0.6431	Age at mer
12	97506357	-0.024	0.004237	1.47E-08	rs7979001	A	G	0.5129	Age at mer

12	1.09E+08	0.028	0.005049	2.93E-08	rs3764002	T	C	0.7406	Age at mer
12	1.11E+08	0.024	0.00432	2.77E-08	rs2106404	C	T	0.4414	Age at mer
13	40238492	0.031	0.004436	2.78E-12	rs9548873	T	C	0.33	Age at mer
13	49475780	-0.033	0.005915	2.42E-08	rs9568123	G	A	0.8509	Age at mer
13	59833252	0.032	0.004527	1.57E-12	rs1327938	C	T	0.3509	Age at mer
13	74600274	-0.034	0.004579	1.13E-13	rs1925047	C	A	0.2942	Age at mer
13	1.12E+08	0.041	0.004441	2.65E-20	rs9522262	G	C	0.4801	Age at mer
14	30501371	0.064	0.011032	6.58E-09	rs1013433	T	C	0.95328	Age at mer
14	60943106	0.054	0.004616	1.31E-31	rs1013891	C	T	0.3072	Age at mer
14	78634174	-0.028	0.005103	4.10E-08	rs213558	A	G	0.2247	Age at mer
14	93909749	0.031	0.004526	7.42E-12	rs1008344	G	A	0.3191	Age at mer
14	1.01E+08	-0.051	0.005201	1.06E-22	rs732898	A	G	0.3082	Age at mer
14	1.01E+08	-0.045	0.008219	4.38E-08	rs7784850	T	G	0.9175	Age at mer
15	23794517	-0.048	0.004612	2.29E-25	rs7178532	A	G	0.3131	Age at mer
15	24540383	0.043	0.007472	8.68E-09	rs2853292	G	A	0.8479	Age at mer
15	47816374	-0.03	0.00471	1.90E-10	rs1107059	A	G	0.6839	Age at mer
15	54362745	0.024	0.004377	4.18E-08	rs1107102	A	G	0.3907	Age at mer
15	60749843	-0.059	0.007171	1.91E-16	rs1727015	G	A	0.9175	Age at mer
15	64625413	0.075	0.012694	3.46E-09	rs1170081	A	G	0.9672	Age at mer
15	67988133	0.042	0.004264	6.84E-23	rs3784692	T	C	0.4076	Age at mer
15	77799657	0.024	0.004307	2.51E-08	rs4886869	G	A	0.4076	Age at mer
15	83299364	-0.029	0.00432	1.90E-11	rs2837446	C	A	0.5934	Age at mer
15	89037134	0.04	0.004319	2.03E-20	rs752278	A	G	0.5875	Age at mer
16	3627358	0.029	0.004828	1.90E-09	rs758747	T	C	0.7197	Age at mer
16	14388750	-0.047	0.004485	1.07E-25	rs1704528	C	T	0.6531	Age at mer
16	19751210	-0.043	0.006493	3.54E-11	rs7276953	C	T	0.8807	Age at mer
16	20375776	-0.025	0.004273	4.89E-09	rs4483850	T	A	0.5408	Age at mer
16	29880874	0.042	0.004923	1.44E-17	rs3533097	T	C	0.6998	Age at mer
16	53814363	0.049	0.004311	6.23E-30	rs9972653	T	G	0.5865	Age at mer
16	69733460	0.053	0.004248	1.00E-35	rs7359336	A	G	0.4245	Age at mer
17	6034754	0.035	0.005068	4.97E-12	rs1260328	A	G	0.7624	Age at mer
17	7774047	-0.046	0.00836	3.74E-08	rs5568096	G	A	0.93042	Age at mer
17	43173273	-0.025	0.004352	9.24E-09	rs2301597	C	T	0.4911	Age at mer
17	49613785	-0.059	0.004637	4.39E-37	rs9635759	A	G	0.7048	Age at mer
17	53229345	-0.033	0.004392	5.76E-14	rs1994234	G	A	0.4105	Age at mer
17	78779665	0.032	0.005166	5.86E-10	rs5915309	C	A	0.7724	Age at mer
18	3813464	0.051	0.004816	3.32E-26	rs1187390	A	G	0.2823	Age at mer
18	44785302	-0.053	0.004257	1.40E-35	rs2668767	C	T	0.4026	Age at mer
19	1861023	-0.027	0.004391	7.82E-10	rs5628964	A	G	0.4682	Age at mer
19	4980864	-0.029	0.004562	2.05E-10	rs169080	C	T	0.3708	Age at mer
19	7891514	-0.03	0.005167	6.38E-09	rs610445	G	C	0.2406	Age at mer
19	10006795	-0.046	0.004366	5.95E-26	rs7256078	T	C	0.5417	Age at mer
19	18819060	-0.036	0.005266	8.16E-12	rs6002178	A	T	0.7604	Age at mer
19	31044844	0.032	0.005869	4.97E-08	rs1167313	C	G	0.827	Age at mer
19	34311481	0.028	0.004478	4.03E-10	rs29938	C	T	0.3449	Age at mer

19	36204690	-0.029	0.004138	2.40E-12	rs107068	G	A	0.5209	Age at mei
19	47609223	0.044	0.0047	7.89E-21	rs4804025	A	G	0.3131	Age at mei
19	49209339	-0.024	0.004374	4.09E-08	rs2548459	C	T	0.5298	Age at mei
20	17109159	-0.035	0.004312	4.77E-16	rs852061	C	A	0.3817	Age at mei
20	20348962	0.036	0.00622	7.12E-09	rs1115583	T	C	0.8857	Age at mei
20	33456921	-0.031	0.00499	5.23E-10	rs7273470	C	G	0.8101	Age at mei
20	37297776	-0.035	0.005151	1.08E-11	rs6027163	A	G	0.7604	Age at mei
20	54823550	0.043	0.00678	2.26E-10	rs1157588	G	A	0.8767	Age at mei
20	62443239	-0.025	0.004487	2.52E-08	rs6011149	A	G	0.6382	Age at mei
21	37768628	-0.044	0.006522	1.52E-11	rs6222940	A	G	0.8588	Age at mei
21	40611442	0.032	0.004462	7.41E-13	rs4818008	A	T	0.6024	Age at mei
22	22162374	0.027	0.004204	1.34E-10	rs5749998	T	A	0.4771	Age at mei
22	31287711	0.032	0.004697	9.61E-12	rs1170556	A	G	0.7256	Age at mei
22	49678782	0.035	0.004848	5.23E-13	rs8136272	T	A	0.7485	Age at mei
6	1.06E+08	1.39652	0.303451	4.18E-06	rs1266374	T	C	0.065423	Diabetes o
8	84886337	2.25694	0.448782	4.93E-07	rs7675205	G	A	0.035588	Diabetes o
9	1.02E+08	2.24545	0.418003	7.79E-08	rs7671520	T	C	0.038279	Diabetes o
9	1.04E+08	0.899672	0.18758	1.62E-06	rs7274339	C	T	0.178398	Diabetes o
11	35565554	0.984161	0.215159	4.78E-06	rs7783474	C	T	0.137503	Diabetes o
16	79108940	0.653982	0.140948	3.49E-06	rs9937803	G	A	0.418518	Diabetes o
17	59518015	4.27399	0.844686	4.20E-07	rs1469446	A	G	0.01177	Diabetes o
1	94457196	1.3	0.26	6.40E-07	rs1711071	T	A	0.031	Illnesses o
1	2.12E+08	0.83	0.18	3.60E-06	rs1214328	T	C	0.061	Illnesses o
3	1.15E+08	1.6	0.33	2.90E-06	rs7913503	G	A	0.02	Illnesses o
4	1.13E+08	0.44	0.097	4.30E-06	rs1704409	T	G	0.24	Illnesses o
6	1.55E+08	0.74	0.15	1.90E-06	rs3930218	G	A	0.083	Illnesses o
12	26922895	0.49	0.1	3.00E-06	rs1050601	C	T	0.8	Illnesses o
12	1.26E+08	-0.43	0.093	3.00E-06	rs6194111	A	G	0.27	Illnesses o
22	27381298	0.77	0.14	3.40E-08	rs9613318	A	G	0.11	Illnesses o
1	44097438	-0.007673	0.001222	3.39E-10	rs1240597	T	G	0.347724	Maternal s
4	1.41E+08	-0.007357	0.001213	1.30E-09	rs1731480	T	C	0.360416	Maternal s
6	26159356	-0.008303	0.001393	2.50E-09	rs2183947	A	G	0.224611	Maternal s
8	92990029	-0.008132	0.001435	1.47E-08	rs2028652	C	T	0.228674	Maternal s
9	1.36E+08	0.012692	0.001854	7.64E-12	rs7559618	T	C	0.111233	Maternal s
15	78862762	-0.01034	0.001416	2.79E-13	rs664172	A	G	0.220438	Maternal s
20	61986949	0.012569	0.001659	3.51E-14	rs2273500	C	T	0.144147	Maternal s
1	3675002	0.015869	0.00322	8.33E-07	rs6682863	A	G	0.935965	Maternal s
1	51039654	0.017543	0.003306	1.12E-07	rs6669941	A	G	0.935605	Maternal s
1	2.15E+08	0.010602	0.002288	3.60E-06	rs3509078	T	C	0.86209	Maternal s
2	338364	0.007706	0.001602	1.52E-06	rs2203063	T	C	0.571303	Maternal s
2	1.65E+08	-0.008394	0.001783	2.52E-06	rs3556616	G	A	0.723995	Maternal s
3	51921338	0.033453	0.006691	5.74E-07	rs1442002	A	G	0.985124	Maternal s
3	68655281	-0.007669	0.00166	3.87E-06	rs4855340	C	T	0.662177	Maternal s
3	1.53E+08	0.008736	0.001635	9.14E-08	rs9874232	C	A	0.633656	Maternal s
4	96021973	0.00784	0.001636	1.65E-06	rs1343464	G	A	0.638435	Maternal s

4	1.41E+08	0.007735	0.001619	1.78E-06	rs3561272	A	G	0.616756	Maternal s
4	1.44E+08	0.035074	0.006782	2.33E-07	rs1507913	C	T	0.9853	Maternal s
5	1.04E+08	0.010142	0.002003	4.12E-07	rs7461386	C	G	0.810427	Maternal s
6	26159356	0.009898	0.001876	1.33E-07	rs2183947	A	G	0.775427	Maternal s
7	4948043	-0.009347	0.001996	2.83E-06	rs5637416	C	G	0.786963	Maternal s
7	79884394	0.017847	0.003428	1.93E-07	rs306697	A	G	0.943186	Maternal s
7	1.37E+08	0.007477	0.001617	3.78E-06	rs322301	C	A	0.615097	Maternal s
8	16694516	0.00855	0.001871	4.91E-06	rs6999072	A	C	0.6987	Maternal s
8	70914300	-0.008964	0.001866	1.56E-06	rs304600	C	A	0.768085	Maternal s
8	93111532	-0.009579	0.001909	5.22E-07	rs1484559	C	T	0.78249	Maternal s
10	95595983	-0.028148	0.005781	1.12E-06	rs1414509	T	C	0.978629	Maternal s
10	1.07E+08	-0.008079	0.001758	4.30E-06	rs2491365	C	T	0.723487	Maternal s
11	29878815	0.014737	0.00299	8.26E-07	rs5631863	A	C	0.924511	Maternal s
11	1.14E+08	-0.009921	0.001835	6.41E-08	rs1633547	T	C	0.75882	Maternal s
12	23990983	-0.011634	0.002474	2.58E-06	rs9668905	A	T	0.881608	Maternal s
12	50015942	0.014389	0.002984	1.42E-06	rs7408691	A	G	0.925188	Maternal s
12	1.18E+08	0.019106	0.004183	4.93E-06	rs6193872	T	C	0.958914	Maternal s
15	78870803	0.011467	0.001875	9.58E-10	rs576982	T	C	0.773641	Maternal s
16	88921493	-0.008022	0.001756	4.92E-06	rs1293258	A	C	0.724108	Maternal s
18	1837007	-0.011438	0.002279	5.21E-07	rs1371274	C	A	0.863287	Maternal s
20	50348811	-0.025661	0.005534	3.54E-06	rs7313565	A	G	0.97867	Maternal s
20	61986949	-0.01195	0.00224	9.61E-08	rs2273500	C	T	0.85632	Maternal s
1	44140075	-0.036768	0.005472	1.83E-11	rs1078944	C	A	0.333704	Maternal s
4	1.41E+08	-0.034899	0.005304	4.73E-11	rs3607264	A	T	0.381357	Maternal s
6	26159356	-0.037155	0.006143	1.47E-09	rs2183947	A	G	0.225156	Maternal s
7	32315613	0.032439	0.005326	1.12E-09	rs1022622	G	A	0.369832	Maternal s
8	93114414	-0.038054	0.006288	1.44E-09	rs7002049	T	C	0.214316	Maternal s
9	1.36E+08	0.058657	0.008236	1.07E-12	rs1139274	G	C	0.110208	Maternal s
10	1.05E+08	0.041761	0.007379	1.52E-08	rs1088380	G	A	0.140893	Maternal s
15	78870803	-0.045361	0.006135	1.43E-13	rs576982	T	C	0.227239	Maternal s
16	24830866	-0.032253	0.005796	2.62E-08	rs1292094	A	T	0.273445	Maternal s
17	32906373	0.034781	0.006297	3.33E-08	rs5632237	T	G	0.230467	Maternal s
20	61984317	0.049234	0.006548	5.53E-14	rs6011779	C	T	0.190062	Maternal s

mr_keep.e	pval_origi	id.exposur	data_sour	samplesize	r2	F
TRUE	reported	3UJN2L	textfile	418758	7.59E-05	31.78797
TRUE	reported	3UJN2L	textfile	418758	0.000124	51.75978
TRUE	reported	3UJN2L	textfile	418758	8.41E-05	35.20428
TRUE	reported	3UJN2L	textfile	418758	8.01E-05	33.5471
TRUE	reported	3UJN2L	textfile	418758	8.72E-05	36.53753
TRUE	reported	3UJN2L	textfile	418758	8.07E-05	33.7778
TRUE	reported	3UJN2L	textfile	418758	8.04E-05	33.65521
TRUE	reported	3UJN2L	textfile	418758	9.67E-05	40.49567
TRUE	reported	3UJN2L	textfile	418758	8.43E-05	35.29741
TRUE	reported	3UJN2L	textfile	418758	8.85E-05	37.056
TRUE	reported	3UJN2L	textfile	418758	0.000116	48.45229
TRUE	reported	3UJN2L	textfile	418758	7.93E-05	33.20389
TRUE	reported	3UJN2L	textfile	418758	7.24E-05	30.31952
TRUE	reported	3UJN2L	textfile	418758	0.00014	58.52965
TRUE	reported	3UJN2L	textfile	418758	7.71E-05	32.2829
TRUE	reported	3UJN2L	textfile	418758	7.19E-05	30.12776
TRUE	reported	3UJN2L	textfile	418758	7.49E-05	31.37516
TRUE	reported	3UJN2L	textfile	418758	8.03E-05	33.63984
TRUE	reported	3UJN2L	textfile	418758	7.88E-05	32.99338
TRUE	reported	3UJN2L	textfile	418758	8.71E-05	36.47195
TRUE	reported	3UJN2L	textfile	418758	0.000337	141.162
TRUE	reported	3UJN2L	textfile	418758	9.21E-05	38.57045
TRUE	reported	3UJN2L	textfile	418758	0.000101	42.38392
TRUE	reported	3UJN2L	textfile	418758	0.000122	51.0598
TRUE	reported	3UJN2L	textfile	418758	7.54E-05	31.55781
TRUE	reported	3UJN2L	textfile	418758	0.000103	43.02948
TRUE	reported	3UJN2L	textfile	418758	7.37E-05	30.85183
TRUE	reported	3UJN2L	textfile	418758	8.15E-05	34.15184
TRUE	reported	3UJN2L	textfile	418758	7.17E-05	30.0403
TRUE	reported	3UJN2L	textfile	418758	9.42E-05	39.44291
TRUE	reported	3UJN2L	textfile	418758	7.43E-05	31.10192
TRUE	reported	3UJN2L	textfile	418758	0.000166	69.39019
TRUE	reported	3UJN2L	textfile	418758	8.98E-05	37.59858
TRUE	reported	3UJN2L	textfile	418758	8.50E-05	35.5942
TRUE	reported	3UJN2L	textfile	418758	8.49E-05	35.53532
TRUE	reported	3UJN2L	textfile	418758	7.80E-05	32.66891
TRUE	reported	3UJN2L	textfile	418758	8.41E-05	35.20428
TRUE	reported	3UJN2L	textfile	418758	9.46E-05	39.62983
TRUE	reported	3UJN2L	textfile	418758	9.18E-05	38.43982
TRUE	reported	3UJN2L	textfile	418758	0.000107	44.87678
TRUE	reported	3UJN2L	textfile	418758	9.48E-05	39.68981
TRUE	reported	3UJN2L	textfile	418758	0.000122	50.8984
TRUE	reported	3UJN2L	textfile	418758	9.34E-05	39.12883

TRUE	reported	3UJN2L	textfile	418758	7.16E-05	29.98852
TRUE	reported	3UJN2L	textfile	418758	7.87E-05	32.94012
TRUE	reported	3UJN2L	textfile	418758	8.23E-05	34.44686
TRUE	reported	3UJN2L	textfile	418758	0.000127	53.02462
TRUE	reported	3UJN2L	textfile	418758	8.25E-05	34.53196
TRUE	reported	3UJN2L	textfile	418758	8.67E-05	36.32269
TRUE	reported	3UJN2L	textfile	418758	0.000112	47.00677
TRUE	reported	3UJN2L	textfile	418758	8.28E-05	34.65609
TRUE	reported	3UJN2L	textfile	418758	7.26E-05	30.40142
TRUE	reported	3UJN2L	textfile	418758	7.20E-05	30.13772
TRUE	reported	3UJN2L	textfile	418758	7.43E-05	31.11146
TRUE	reported	3UJN2L	textfile	418758	9.15E-05	38.31694
TRUE	reported	3UJN2L	textfile	418758	7.30E-05	30.55618
TRUE	reported	3UJN2L	textfile	418758	8.48E-05	35.51823
TRUE	reported	3UJN2L	textfile	418758	8.97E-05	37.54548
TRUE	reported	3UJN2L	textfile	418758	9.95E-05	41.68375
TRUE	reported	3UJN2L	textfile	418758	8.00E-05	33.51225
TRUE	reported	3UJN2L	textfile	418758	7.76E-05	32.49882
TRUE	reported	3UJN2L	textfile	418758	0.000129	53.81861
TRUE	reported	3UJN2L	textfile	418758	0.000103	43.21824
TRUE	reported	3UJN2L	textfile	418758	7.20E-05	30.13996
TRUE	reported	3UJN2L	textfile	418758	7.12E-05	29.8217
TRUE	reported	3UJN2L	textfile	418758	7.54E-05	31.55781
TRUE	reported	qOy15B	textfile	397338	8.80E-05	34.9786
TRUE	reported	qOy15B	textfile	397338	0.000254	100.9541
TRUE	reported	qOy15B	textfile	397338	9.50E-05	37.7345
TRUE	reported	qOy15B	textfile	397338	8.77E-05	34.86604
TRUE	reported	qOy15B	textfile	397338	0.000123	48.99975
TRUE	reported	qOy15B	textfile	397338	0.00012	47.6755
TRUE	reported	qOy15B	textfile	397338	6.90E-05	27.4375
TRUE	reported	qOy15B	textfile	397338	8.56E-05	34.02761
TRUE	reported	qOy15B	textfile	397338	7.23E-05	28.72946
TRUE	reported	qOy15B	textfile	397338	7.74E-05	30.75191
TRUE	reported	qOy15B	textfile	397338	0.000165	65.53255
TRUE	reported	qOy15B	textfile	397338	9.34E-05	37.1336
TRUE	reported	qOy15B	textfile	397338	9.18E-05	36.48142
TRUE	reported	qOy15B	textfile	397338	8.35E-05	33.19938
TRUE	reported	qOy15B	textfile	397338	7.81E-05	31.04066
TRUE	reported	qOy15B	textfile	397338	7.66E-05	30.45389
TRUE	reported	qOy15B	textfile	397338	0.000279	110.75
TRUE	reported	qOy15B	textfile	397338	0.000187	74.28761
TRUE	reported	qOy15B	textfile	397338	8.63E-05	34.30595
TRUE	reported	qOy15B	textfile	397338	0.000125	49.66868
TRUE	reported	qOy15B	textfile	397338	7.95E-05	31.57354
TRUE	reported	qOy15B	textfile	397338	9.41E-05	37.39774

TRUE	reported	qOy15B	textfile	397338	0.000136	54.22287
TRUE	reported	qOy15B	textfile	397338	7.84E-05	31.17345
TRUE	reported	qOy15B	textfile	397338	9.06E-05	35.99982
TRUE	reported	qOy15B	textfile	397338	0.000109	43.46206
TRUE	reported	qOy15B	textfile	397338	0.000107	42.55988
TRUE	reported	qOy15B	textfile	397338	0.000153	60.98836
TRUE	reported	qOy15B	textfile	397338	9.92E-05	39.4382
TRUE	reported	qOy15B	textfile	397338	7.95E-05	31.57354
TRUE	reported	qOy15B	textfile	397338	0.000208	82.72294
TRUE	reported	qOy15B	textfile	397338	7.89E-05	31.35984
TRUE	reported	qOy15B	textfile	397338	0.000155	61.73438
TRUE	reported	qOy15B	textfile	397338	0.000118	47.02017
TRUE	reported	qOy15B	textfile	397338	8.13E-05	32.32117
TRUE	reported	qOy15B	textfile	397338	0.000113	45.08141
TRUE	reported	qOy15B	textfile	397338	9.06E-05	35.99982
TRUE	reported	qOy15B	textfile	397338	8.22E-05	32.6529
TRUE	reported	qOy15B	textfile	397338	0.000115	45.8696
TRUE	reported	qOy15B	textfile	397338	7.95E-05	31.57354
TRUE	reported	qOy15B	textfile	397338	0.000122	48.33536
TRUE	reported	qOy15B	textfile	397338	7.77E-05	30.86404
TRUE	reported	qOy15B	textfile	397338	0.000252	99.9995
TRUE	reported	qOy15B	textfile	397338	0.000224	89.01467
TRUE	reported	qOy15B	textfile	397338	0.000299	119.0077
TRUE	reported	qOy15B	textfile	397338	0.0001	39.82025
TRUE	reported	qOy15B	textfile	397338	7.67E-05	30.47025
TRUE	reported	qOy15B	textfile	397338	0.000188	74.6986
TRUE	reported	qOy15B	textfile	397338	0.000171	67.89726
TRUE	reported	qOy15B	textfile	397338	0.00011	43.76309
TRUE	reported	qOy15B	textfile	397338	0.000112	44.44422
TRUE	reported	qOy15B	textfile	397338	0.000112	44.44422
TRUE	reported	qOy15B	textfile	397338	0.000112	44.44422
TRUE	reported	qOy15B	textfile	397338	0.000185	73.46902
TRUE	reported	qOy15B	textfile	397338	9.06E-05	35.99982
TRUE	reported	qOy15B	textfile	397338	0.000105	41.70986
TRUE	reported	qOy15B	textfile	397338	7.74E-05	30.75191
TRUE	reported	qOy15B	textfile	397338	9.57E-05	38.02759
TRUE	reported	qOy15B	textfile	397338	7.55E-05	29.98851
TRUE	reported	qOy15B	textfile	397338	0.000105	41.78678
TRUE	reported	qOy15B	textfile	397338	9.92E-05	39.4382
TRUE	reported	qOy15B	textfile	397338	0.00017	67.6046
TRUE	reported	qOy15B	textfile	397338	0.000141	55.89314
TRUE	reported	qOy15B	textfile	397338	7.43E-05	29.53671
TRUE	reported	qOy15B	textfile	397338	0.000106	41.94083
TRUE	reported	qOy15B	textfile	397338	0.000226	89.79773
TRUE	reported	qOy15B	textfile	397338	0.000166	65.78979

TRUE	reported	qOy15B	textfile	397338	0.0001	39.91922
TRUE	reported	qOy15B	textfile	397338	0.000118	46.69421
TRUE	reported	qOy15B	textfile	397338	7.79E-05	30.97149
TRUE	reported	qOy15B	textfile	397338	9.48E-05	37.67401
TRUE	reported	qOy15B	textfile	397338	9.06E-05	35.99982
TRUE	reported	qOy15B	textfile	397338	0.000126	50.17336
TRUE	reported	qOy15B	textfile	397338	8.94E-05	35.52142
TRUE	reported	qOy15B	textfile	397338	9.35E-05	37.15174
TRUE	reported	qOy15B	textfile	397338	0.000152	60.24686
TRUE	reported	qOy15B	textfile	397338	0.000115	45.8696
TRUE	reported	qOy15B	textfile	397338	0.000431	171.4844
TRUE	reported	qOy15B	textfile	397338	7.30E-05	28.99394
TRUE	reported	qOy15B	textfile	397338	0.000104	41.32632
TRUE	reported	qOy15B	textfile	397338	0.000183	72.6977
TRUE	reported	qOy15B	textfile	397338	8.32E-05	33.06233
TRUE	reported	qOy15B	textfile	397338	0.000128	50.76537
TRUE	reported	qOy15B	textfile	397338	9.98E-05	39.64315
TRUE	reported	qOy15B	textfile	397338	8.63E-05	34.30595
TRUE	reported	qOy15B	textfile	397338	9.35E-05	37.15174
TRUE	reported	qOy15B	textfile	397338	7.65E-05	30.41674
TRUE	reported	qOy15B	textfile	397338	8.92E-05	35.45643
TRUE	reported	qOy15B	textfile	397338	0.000153	60.73216
TRUE	reported	qOy15B	textfile	397338	0.000219	87.11067
TRUE	reported	qOy15B	textfile	397338	0.000105	41.66095
TRUE	reported	qOy15B	textfile	397338	0.000105	41.70986
TRUE	reported	qOy15B	textfile	397338	0.000133	52.77569
TRUE	reported	qOy15B	textfile	397338	0.000106	42.24979
TRUE	reported	qOy15B	textfile	397338	0.000115	45.72313
TRUE	reported	qOy15B	textfile	397338	0.000103	41.11052
TRUE	reported	qOy15B	textfile	397338	0.000142	56.60742
TRUE	reported	qOy15B	textfile	397338	0.000144	57.02825
TRUE	reported	qOy15B	textfile	397338	9.83E-05	39.0623
TRUE	reported	qOy15B	textfile	397338	7.68E-05	30.51232
TRUE	reported	qOy15B	textfile	397338	0.000114	45.41565
TRUE	reported	qOy15B	textfile	397338	0.000111	43.95177
TRUE	reported	qOy15B	textfile	397338	7.94E-05	31.53239
TRUE	reported	qOy15B	textfile	397338	8.63E-05	34.30595
TRUE	reported	qOy15B	textfile	397338	8.54E-05	33.94312
TRUE	reported	qOy15B	textfile	397338	0.000189	75.11073
TRUE	reported	qOy15B	textfile	397338	9.79E-05	38.91364
TRUE	reported	qOy15B	textfile	397338	9.79E-05	38.91364
TRUE	reported	qOy15B	textfile	397338	8.20E-05	32.58491
TRUE	reported	qOy15B	textfile	397338	0.000104	41.32632
TRUE	reported	qOy15B	textfile	397338	8.63E-05	34.30595
TRUE	reported	qOy15B	textfile	397338	8.49E-05	33.7504

TRUE	reported	qOy15B	textfile	397338	9.37E-05	37.25189
TRUE	reported	qOy15B	textfile	397338	9.19E-05	36.50155
TRUE	reported	qOy15B	textfile	397338	9.06E-05	35.99982
TRUE	reported	qOy15B	textfile	397338	8.65E-05	34.38206
TRUE	reported	qOy15B	textfile	397338	0.000119	47.10927
TRUE	reported	qOy15B	textfile	397338	7.61E-05	30.24985
TRUE	reported	qOy15B	textfile	397338	8.79E-05	34.91718
TRUE	reported	qOy15B	textfile	397338	8.12E-05	32.2829
TRUE	reported	qOy15B	textfile	397338	0.000108	42.84276
TRUE	reported	qOy15B	textfile	397338	0.00011	43.81157
TRUE	reported	qOy15B	textfile	397338	8.92E-05	35.43066
TRUE	reported	qOy15B	textfile	397338	7.32E-05	29.08276
TRUE	reported	qOy15B	textfile	397338	0.000155	61.73438
TRUE	reported	qOy15B	textfile	397338	7.81E-05	31.04066
TRUE	reported	qOy15B	textfile	397338	0.000134	53.16814
TRUE	reported	qOy15B	textfile	397338	7.50E-05	29.79325
TRUE	reported	qOy15B	textfile	397338	8.85E-05	35.14778
TRUE	reported	qOy15B	textfile	397338	0.000123	48.99975
TRUE	reported	qOy15B	textfile	397338	9.62E-05	38.21468
TRUE	reported	qOy15B	textfile	397338	0.000224	88.89751
TRUE	reported	qOy15B	textfile	397338	0.000104	41.40622
TRUE	reported	qOy15B	textfile	397338	0.00013	51.70269
TRUE	reported	qOy15B	textfile	397338	0.000102	40.49566
TRUE	reported	qOy15B	textfile	397338	8.39E-05	33.32421
TRUE	reported	qOy15B	textfile	397338	9.94E-05	39.51001
TRUE	reported	qOy15B	textfile	397338	0.000135	53.77751
TRUE	reported	qOy15B	textfile	397338	0.000135	53.55552
TRUE	reported	qOy15B	textfile	397338	0.000104	41.32632
TRUE	reported	qOy15B	textfile	397338	0.000132	52.38976
TRUE	reported	qOy15B	textfile	397338	0.000111	44.2911
TRUE	reported	qOy15B	textfile	397338	0.000103	40.95979
TRUE	reported	qOy15B	textfile	397338	9.34E-05	37.09899
TRUE	reported	qOy15B	textfile	397338	8.08E-05	32.11095
TRUE	reported	qOy15B	textfile	397338	8.35E-05	33.19938
TRUE	reported	qOy15B	textfile	397338	0.000115	45.8696
TRUE	reported	qOy15B	textfile	397338	0.000111	44.00089
TRUE	reported	qOy15B	textfile	397338	8.12E-05	32.2829
TRUE	reported	qOy15B	textfile	397338	9.35E-05	37.15174
TRUE	reported	qOy15B	textfile	397338	7.42E-05	29.46924
TRUE	reported	qOy15B	textfile	397338	0.00012	47.78993
TRUE	reported	qOy15B	textfile	397338	0.000131	52.23321
TRUE	reported	qOy15B	textfile	397338	0.000152	60.36064
TRUE	reported	qOy15B	textfile	397338	0.000102	40.71635
TRUE	reported	qOy15B	textfile	397338	0.000161	63.99968
TRUE	reported	qOy15B	textfile	397338	9.48E-05	37.65477

TRUE	reported	qOy15B	textfile	397338	7.66E-05	30.42197
TRUE	reported	qOy15B	textfile	397338	0.000125	49.66868
TRUE	reported	qOy15B	textfile	397338	7.95E-05	31.60247
TRUE	reported	qOy15B	textfile	397338	0.000139	55.1834
TRUE	reported	qOy15B	textfile	397338	9.57E-05	38.02759
TRUE	reported	qOy15B	textfile	397338	0.000169	67.08356
TRUE	reported	qOy15B	textfile	397338	0.000207	82.44599
TRUE	reported	qOy15B	textfile	397338	8.32E-05	33.06233
TRUE	reported	qOy15B	textfile	397338	9.19E-05	36.52345
TRUE	reported	qOy15B	textfile	397338	8.79E-05	34.94106
TRUE	reported	qOy15B	textfile	397338	0.000107	42.51019
TRUE	reported	qOy15B	textfile	397338	6.97E-05	27.67661
TRUE	reported	qOy15B	textfile	397338	0.000122	48.33536
TRUE	reported	qOy15B	textfile	397338	0.000151	59.93726
TRUE	reported	qOy15B	textfile	397338	0.000161	63.99968
TRUE	reported	qOy15B	textfile	397338	8.96E-05	35.60093
TRUE	reported	qOy15B	textfile	397338	0.000155	61.73438
TRUE	reported	qOy15B	textfile	397338	7.29E-05	28.9545
TRUE	reported	qOy15B	textfile	397338	0.000106	41.96765
TRUE	reported	qOy15B	textfile	397338	9.50E-05	37.7345
TRUE	reported	qOy15B	textfile	397338	8.92E-05	35.43066
TRUE	reported	qOy15B	textfile	397338	0.000163	64.64127
TRUE	reported	qOy15B	textfile	397338	0.000115	45.72313
TRUE	reported	qOy15B	textfile	397338	8.21E-05	32.60995
TRUE	reported	qOy15B	textfile	397338	0.000122	48.36546
TRUE	reported	qOy15B	textfile	397338	8.92E-05	35.43066
TRUE	reported	qOy15B	textfile	397338	7.87E-05	31.25811
TRUE	reported	qOy15B	textfile	397338	8.49E-05	33.7504
TRUE	reported	qOy15B	textfile	397338	7.74E-05	30.75191
TRUE	reported	qOy15B	textfile	397338	8.83E-05	35.08266
TRUE	reported	qOy15B	textfile	397338	7.79E-05	30.94125
TRUE	reported	qOy15B	textfile	397338	9.39E-05	37.29701
TRUE	reported	qOy15B	textfile	397338	7.61E-05	30.24985
TRUE	reported	mrqKhx	textfile	182416	0.000183	33.45816
TRUE	reported	mrqKhx	textfile	182416	0.000198	36.06302
TRUE	reported	mrqKhx	textfile	182416	0.000165	30.027
TRUE	reported	mrqKhx	textfile	182416	0.000309	56.31255
TRUE	reported	mrqKhx	textfile	182416	0.00017	31.02258
TRUE	reported	mrqKhx	textfile	182416	0.000365	66.6387
TRUE	reported	mrqKhx	textfile	182416	0.000346	63.15497
TRUE	reported	mrqKhx	textfile	182416	0.000657	119.9327
TRUE	reported	mrqKhx	textfile	182416	0.000288	52.5143
TRUE	reported	mrqKhx	textfile	182416	0.000311	56.78502
TRUE	reported	mrqKhx	textfile	182416	0.00018	32.85847
TRUE	reported	mrqKhx	textfile	182416	0.000202	36.92082

TRUE	reported	mrqKhx	textfile	182416	0.00088	160.6602
TRUE	reported	mrqKhx	textfile	182416	0.000914	166.8523
TRUE	reported	mrqKhx	textfile	182416	0.000284	51.73507
TRUE	reported	mrqKhx	textfile	182416	0.000183	33.46887
TRUE	reported	mrqKhx	textfile	182416	0.000499	91.02216
TRUE	reported	mrqKhx	textfile	182416	0.000882	161.0016
TRUE	reported	mrqKhx	textfile	182416	0.00023	41.88056
TRUE	reported	mrqKhx	textfile	182416	0.000164	29.91212
TRUE	reported	mrqKhx	textfile	182416	0.000863	157.5903
TRUE	reported	mrqKhx	textfile	182416	0.000163	29.73203
TRUE	reported	mrqKhx	textfile	182416	0.000252	45.9545
TRUE	reported	mrqKhx	textfile	182416	0.000194	35.38867
TRUE	reported	mrqKhx	textfile	182416	0.000379	69.22306
TRUE	reported	mrqKhx	textfile	182416	0.000551	100.5739
TRUE	reported	mrqKhx	textfile	182416	0.000272	49.63638
TRUE	reported	mrqKhx	textfile	182416	0.000183	33.34403
TRUE	reported	mrqKhx	textfile	182416	0.000843	153.8384
TRUE	reported	mrqKhx	textfile	182416	0.000193	35.26975
TRUE	reported	mrqKhx	textfile	182416	0.000272	49.69013
TRUE	reported	mrqKhx	textfile	182416	0.00075	136.8956
TRUE	reported	mrqKhx	textfile	182416	0.00021	38.32427
TRUE	reported	mrqKhx	textfile	182416	0.000275	50.22573
TRUE	reported	mrqKhx	textfile	182416	0.000358	65.24255
TRUE	reported	mrqKhx	textfile	182416	0.000229	41.821
TRUE	reported	mrqKhx	textfile	182416	0.000335	61.20168
TRUE	reported	mrqKhx	textfile	182416	0.000489	89.25438
TRUE	reported	mrqKhx	textfile	182416	0.000191	34.84476
TRUE	reported	mrqKhx	textfile	182416	0.000469	85.5396
TRUE	reported	mrqKhx	textfile	182416	0.000567	103.5481
TRUE	reported	mrqKhx	textfile	182416	0.000176	32.1458
TRUE	reported	mrqKhx	textfile	182416	0.000215	39.17694
TRUE	reported	mrqKhx	textfile	182416	0.000874	159.478
TRUE	reported	mrqKhx	textfile	182416	0.000267	48.80251
TRUE	reported	mrqKhx	textfile	182416	0.000175	31.97675
TRUE	reported	mrqKhx	textfile	182416	0.000241	44.01372
TRUE	reported	mrqKhx	textfile	182416	0.000327	59.74398
TRUE	reported	mrqKhx	textfile	182416	0.000168	30.65612
TRUE	reported	mrqKhx	textfile	182416	0.000544	99.28987
TRUE	reported	mrqKhx	textfile	182416	0.000239	43.59388
TRUE	reported	mrqKhx	textfile	182416	0.000326	59.45628
TRUE	reported	mrqKhx	textfile	182416	0.000486	88.70105
TRUE	reported	mrqKhx	textfile	182416	0.000402	73.28802
TRUE	reported	mrqKhx	textfile	182416	0.000477	86.97226
TRUE	reported	mrqKhx	textfile	182416	0.000187	34.12399
TRUE	reported	mrqKhx	textfile	182416	0.000167	30.4989

TRUE	reported	mrqKhx	textfile	182416	0.000233	42.58386
TRUE	reported	mrqKhx	textfile	182416	0.000171	31.23133
TRUE	reported	mrqKhx	textfile	182416	0.00017	31.0688
TRUE	reported	mrqKhx	textfile	182416	0.000287	52.40693
TRUE	reported	mrqKhx	textfile	182416	0.000251	45.75461
TRUE	reported	mrqKhx	textfile	182416	0.00017	31.0302
TRUE	reported	mrqKhx	textfile	182416	0.000301	54.89662
TRUE	reported	mrqKhx	textfile	182416	0.000624	113.885
TRUE	reported	mrqKhx	textfile	182416	0.000226	41.16323
TRUE	reported	mrqKhx	textfile	182416	0.000237	43.26477
TRUE	reported	mrqKhx	textfile	182416	0.000204	37.1744
TRUE	reported	mrqKhx	textfile	182416	0.000201	36.58663
TRUE	reported	mrqKhx	textfile	182416	0.000283	51.60063
TRUE	reported	mrqKhx	textfile	182416	0.000166	30.36428
TRUE	reported	mrqKhx	textfile	182416	0.000234	42.75589
TRUE	reported	mrqKhx	textfile	182416	0.000233	42.51293
TRUE	reported	mrqKhx	textfile	182416	0.000423	77.11767
TRUE	reported	mrqKhx	textfile	182416	0.000379	69.10124
TRUE	reported	mrqKhx	textfile	182416	0.000292	53.30841
TRUE	reported	mrqKhx	textfile	182416	0.000293	53.5192
TRUE	reported	mrqKhx	textfile	182416	0.000185	33.7831
TRUE	reported	mrqKhx	textfile	182416	0.000326	59.48801
TRUE	reported	mrqKhx	textfile	182416	0.000515	94.07263
TRUE	reported	mrqKhx	textfile	182416	0.000273	49.73421
TRUE	reported	mrqKhx	textfile	182416	0.000225	41.04146
TRUE	reported	mrqKhx	textfile	182416	0.003933	720.2105
TRUE	reported	mrqKhx	textfile	182416	0.000458	83.50268
TRUE	reported	mrqKhx	textfile	182416	0.000193	35.1641
TRUE	reported	mrqKhx	textfile	182416	0.000202	36.84313
TRUE	reported	mrqKhx	textfile	182416	0.000289	52.67727
TRUE	reported	mrqKhx	textfile	182416	0.000731	133.5009
TRUE	reported	mrqKhx	textfile	182416	0.000297	54.13627
TRUE	reported	mrqKhx	textfile	182416	0.000203	37.06897
TRUE	reported	mrqKhx	textfile	182416	0.000191	34.88888
TRUE	reported	mrqKhx	textfile	182416	0.000167	30.49889
TRUE	reported	mrqKhx	textfile	182416	0.000233	42.56088
TRUE	reported	mrqKhx	textfile	182416	0.000234	42.64548
TRUE	reported	mrqKhx	textfile	182416	0.00029	52.82781
TRUE	reported	mrqKhx	textfile	182416	0.000687	125.3657
TRUE	reported	mrqKhx	textfile	182416	0.000179	32.70938
TRUE	reported	mrqKhx	textfile	182416	0.000195	35.6426
TRUE	reported	mrqKhx	textfile	182416	0.000308	56.27475
TRUE	reported	mrqKhx	textfile	182416	0.000283	51.60352
TRUE	reported	mrqKhx	textfile	182416	0.000481	87.82155
TRUE	reported	mrqKhx	textfile	182416	0.000199	36.30149

TRUE	reported	mrqKhx	textfile	182416	0.000342	62.4732
TRUE	reported	mrqKhx	textfile	182416	0.000183	33.35413
TRUE	reported	mrqKhx	textfile	182416	0.000299	54.5178
TRUE	reported	mrqKhx	textfile	182416	0.000247	44.98007
TRUE	reported	mrqKhx	textfile	182416	0.000409	74.57007
TRUE	reported	mrqKhx	textfile	182416	0.000169	30.89732
TRUE	reported	mrqKhx	textfile	182416	0.000236	42.96903
TRUE	reported	mrqKhx	textfile	182416	0.000179	32.67338
TRUE	reported	mrqKhx	textfile	182416	0.000245	44.66283
TRUE	reported	mrqKhx	textfile	182416	0.000284	51.85622
TRUE	reported	mrqKhx	textfile	182416	0.000488	89.10087
TRUE	reported	mrqKhx	textfile	182416	0.000204	37.28586
TRUE	reported	mrqKhx	textfile	182416	0.002644	483.4945
TRUE	reported	mrqKhx	textfile	182416	0.000951	173.5988
TRUE	reported	mrqKhx	textfile	182416	0.000284	51.89273
TRUE	reported	mrqKhx	textfile	182416	0.000441	80.5176
TRUE	reported	mrqKhx	textfile	182416	0.000169	30.79992
TRUE	reported	mrqKhx	textfile	182416	0.00018	32.89411
TRUE	reported	mrqKhx	textfile	182416	0.000182	33.1296
TRUE	reported	mrqKhx	textfile	182416	0.000169	30.87604
TRUE	reported	mrqKhx	textfile	182416	0.000429	78.21872
TRUE	reported	mrqKhx	textfile	182416	0.000182	33.20082
TRUE	reported	mrqKhx	textfile	182416	0.000223	40.71599
TRUE	reported	mrqKhx	textfile	182416	0.000221	40.37089
TRUE	reported	mrqKhx	textfile	182416	0.000238	43.35268
TRUE	reported	mrqKhx	textfile	182416	0.000214	39.13205
TRUE	reported	mrqKhx	textfile	182416	0.00039	71.19794
TRUE	reported	mrqKhx	textfile	182416	0.000568	103.6604
TRUE	reported	mrqKhx	textfile	182416	0.000207	37.7744
TRUE	reported	mrqKhx	textfile	182416	0.000304	55.4631
TRUE	reported	mrqKhx	textfile	182416	0.000201	36.64077
TRUE	reported	mrqKhx	textfile	182416	0.000206	37.52997
TRUE	reported	mrqKhx	textfile	182416	0.000231	42.19243
TRUE	reported	mrqKhx	textfile	182416	0.000313	57.04417
TRUE	reported	mrqKhx	textfile	182416	0.000195	35.57793
TRUE	reported	mrqKhx	textfile	182416	0.000323	58.87451
TRUE	reported	mrqKhx	textfile	182416	0.00021	38.30482
TRUE	reported	mrqKhx	textfile	182416	0.000179	32.74605
TRUE	reported	mrqKhx	textfile	182416	0.000608	110.958
TRUE	reported	mrqKhx	textfile	182416	0.001121	204.7688
TRUE	reported	mrqKhx	textfile	182416	0.000163	29.75167
TRUE	reported	mrqKhx	textfile	182416	0.000242	44.15049
TRUE	reported	mrqKhx	textfile	182416	0.000198	36.07324
TRUE	reported	mrqKhx	textfile	182416	0.000467	85.30961
TRUE	reported	mrqKhx	textfile	182416	0.000176	32.09219

TRUE	reported	mrqKhx	textfile	182416	0.000169	30.753
TRUE	reported	mrqKhx	textfile	182416	0.000169	30.86197
TRUE	reported	mrqKhx	textfile	182416	0.000268	48.83747
TRUE	reported	mrqKhx	textfile	182416	0.000171	31.12414
TRUE	reported	mrqKhx	textfile	182416	0.000274	49.95836
TRUE	reported	mrqKhx	textfile	182416	0.000302	55.12618
TRUE	reported	mrqKhx	textfile	182416	0.000467	85.23355
TRUE	reported	mrqKhx	textfile	182416	0.000184	33.65479
TRUE	reported	mrqKhx	textfile	182416	0.00075	136.834
TRUE	reported	mrqKhx	textfile	182416	0.000165	30.10123
TRUE	reported	mrqKhx	textfile	182416	0.000257	46.91273
TRUE	reported	mrqKhx	textfile	182416	0.000527	96.15826
TRUE	reported	mrqKhx	textfile	182416	0.000164	29.97313
TRUE	reported	mrqKhx	textfile	182416	0.000593	108.3163
TRUE	reported	mrqKhx	textfile	182416	0.000182	33.11611
TRUE	reported	mrqKhx	textfile	182416	0.000222	40.56643
TRUE	reported	mrqKhx	textfile	182416	0.000165	30.06377
TRUE	reported	mrqKhx	textfile	182416	0.000371	67.69267
TRUE	reported	mrqKhx	textfile	182416	0.000191	34.90569
TRUE	reported	mrqKhx	textfile	182416	0.000532	97.0256
TRUE	reported	mrqKhx	textfile	182416	0.00017	31.05327
TRUE	reported	mrqKhx	textfile	182416	0.000247	45.0707
TRUE	reported	mrqKhx	textfile	182416	0.00047	85.76057
TRUE	reported	mrqKhx	textfile	182416	0.000198	36.07324
TRUE	reported	mrqKhx	textfile	182416	0.000602	109.8244
TRUE	reported	mrqKhx	textfile	182416	0.00024	43.85245
TRUE	reported	mrqKhx	textfile	182416	0.000188	34.23233
TRUE	reported	mrqKhx	textfile	182416	0.000399	72.79201
TRUE	reported	mrqKhx	textfile	182416	0.000708	129.1671
TRUE	reported	mrqKhx	textfile	182416	0.000853	155.6673
TRUE	reported	mrqKhx	textfile	182416	0.000261	47.69826
TRUE	reported	mrqKhx	textfile	182416	0.000166	30.27947
TRUE	reported	mrqKhx	textfile	182416	0.000181	32.99456
TRUE	reported	mrqKhx	textfile	182416	0.000887	161.8802
TRUE	reported	mrqKhx	textfile	182416	0.000309	56.45094
TRUE	reported	mrqKhx	textfile	182416	0.00021	38.36709
TRUE	reported	mrqKhx	textfile	182416	0.000614	112.1444
TRUE	reported	mrqKhx	textfile	182416	0.000849	154.9986
TRUE	reported	mrqKhx	textfile	182416	0.000207	37.80412
TRUE	reported	mrqKhx	textfile	182416	0.000222	40.41797
TRUE	reported	mrqKhx	textfile	182416	0.000185	33.71483
TRUE	reported	mrqKhx	textfile	182416	0.000608	110.9878
TRUE	reported	mrqKhx	textfile	182416	0.000256	46.72642
TRUE	reported	mrqKhx	textfile	182416	0.000163	29.72813
TRUE	reported	mrqKhx	textfile	182416	0.000214	39.09785

TRUE	reported	mrqKhx	textfile	182416	0.000269	49.12577
TRUE	reported	mrqKhx	textfile	182416	0.00048	87.62951
TRUE	reported	mrqKhx	textfile	182416	0.000165	30.10597
TRUE	reported	mrqKhx	textfile	182416	0.000361	65.88848
TRUE	reported	mrqKhx	textfile	182416	0.000184	33.50138
TRUE	reported	mrqKhx	textfile	182416	0.000212	38.58907
TRUE	reported	mrqKhx	textfile	182416	0.000253	46.17719
TRUE	reported	mrqKhx	textfile	182416	0.00022	40.22744
TRUE	reported	mrqKhx	textfile	182416	0.00017	31.04555
TRUE	reported	mrqKhx	textfile	182416	0.000249	45.50772
TRUE	reported	mrqKhx	textfile	182416	0.000282	51.43197
TRUE	reported	mrqKhx	textfile	182416	0.000226	41.24885
TRUE	reported	mrqKhx	textfile	182416	0.000254	46.40591
TRUE	reported	mrqKhx	textfile	182416	0.000286	52.11605
TRUE	reported	hzTFAg	textfile	113	0.157845	20.80471
TRUE	reported	hzTFAg	textfile	113	0.182884	24.84355
TRUE	reported	hzTFAg	textfile	113	0.203422	28.34605
TRUE	reported	hzTFAg	textfile	113	0.169139	22.59643
TRUE	reported	hzTFAg	textfile	113	0.156228	20.55218
TRUE	reported	hzTFAg	textfile	113	0.160029	21.14746
TRUE	reported	hzTFAg	textfile	113	0.184717	25.14901
TRUE	reported	93GFWQ	textfile	408154	6.12E-05	24.99988
TRUE	reported	93GFWQ	textfile	408154	5.21E-05	21.26224
TRUE	reported	93GFWQ	textfile	408154	5.76E-05	23.50769
TRUE	reported	93GFWQ	textfile	408154	5.04E-05	20.57594
TRUE	reported	93GFWQ	textfile	408154	5.96E-05	24.33766
TRUE	reported	93GFWQ	textfile	408154	5.88E-05	24.00988
TRUE	reported	93GFWQ	textfile	408154	5.24E-05	21.37809
TRUE	reported	93GFWQ	textfile	408154	7.41E-05	30.24985
TRUE	reported	gor4ow	textfile	309942	0.000127	39.43843
TRUE	reported	gor4ow	textfile	309942	0.000119	36.80828
TRUE	reported	gor4ow	textfile	309942	0.000115	35.54241
TRUE	reported	gor4ow	textfile	309942	0.000104	32.09746
TRUE	reported	gor4ow	textfile	309942	0.000151	46.85855
TRUE	reported	gor4ow	textfile	309942	0.000172	53.35299
TRUE	reported	gor4ow	textfile	309942	0.000185	57.43061
TRUE	reported	Xa9EyB	textfile	167814	0.000145	24.2816
TRUE	reported	Xa9EyB	textfile	167814	0.000168	28.16407
TRUE	reported	Xa9EyB	textfile	167814	0.000128	21.47036
TRUE	reported	Xa9EyB	textfile	167814	0.000138	23.12497
TRUE	reported	Xa9EyB	textfile	167814	0.000132	22.15332
TRUE	reported	Xa9EyB	textfile	167814	0.000149	24.99888
TRUE	reported	Xa9EyB	textfile	167814	0.000127	21.33053
TRUE	reported	Xa9EyB	textfile	167814	0.00017	28.55059
TRUE	reported	Xa9EyB	textfile	167814	0.000137	22.96279

TRUE	reported	Xa9EyB	textfile	167814	0.000136	22.82465
TRUE	reported	Xa9EyB	textfile	167814	0.000159	26.74219
TRUE	reported	Xa9EyB	textfile	167814	0.000153	25.63728
TRUE	reported	Xa9EyB	textfile	167814	0.000166	27.82738
TRUE	reported	Xa9EyB	textfile	167814	0.000131	21.92902
TRUE	reported	Xa9EyB	textfile	167814	0.000161	27.10531
TRUE	reported	Xa9EyB	textfile	167814	0.000127	21.37282
TRUE	reported	Xa9EyB	textfile	167814	0.000124	20.87383
TRUE	reported	Xa9EyB	textfile	167814	0.000137	23.06961
TRUE	reported	Xa9EyB	textfile	167814	0.00015	25.18155
TRUE	reported	Xa9EyB	textfile	167814	0.000141	23.70977
TRUE	reported	Xa9EyB	textfile	167814	0.000126	21.12582
TRUE	reported	Xa9EyB	textfile	167814	0.000145	24.29711
TRUE	reported	Xa9EyB	textfile	167814	0.000174	29.23652
TRUE	reported	Xa9EyB	textfile	167814	0.000132	22.10577
TRUE	reported	Xa9EyB	textfile	167814	0.000139	23.25193
TRUE	reported	Xa9EyB	textfile	167814	0.000124	20.8654
TRUE	reported	Xa9EyB	textfile	167814	0.000223	37.41357
TRUE	reported	Xa9EyB	textfile	167814	0.000124	20.86879
TRUE	reported	Xa9EyB	textfile	167814	0.00015	25.18609
TRUE	reported	Xa9EyB	textfile	167814	0.000128	21.49858
TRUE	reported	Xa9EyB	textfile	167814	0.00017	28.45337
TRUE	reported	5PiVJS	textfile	391992	0.000115	45.1461
TRUE	reported	5PiVJS	textfile	391992	0.00011	43.28546
TRUE	reported	5PiVJS	textfile	391992	9.33E-05	36.57891
TRUE	reported	5PiVJS	textfile	391992	9.46E-05	37.10228
TRUE	reported	5PiVJS	textfile	391992	9.34E-05	36.61905
TRUE	reported	5PiVJS	textfile	391992	0.000129	50.72015
TRUE	reported	5PiVJS	textfile	391992	8.17E-05	32.02866
TRUE	reported	5PiVJS	textfile	391992	0.000139	54.66
TRUE	reported	5PiVJS	textfile	391992	7.90E-05	30.96692
TRUE	reported	5PiVJS	textfile	391992	7.78E-05	30.50643
TRUE	reported	5PiVJS	textfile	391992	0.000144	56.53142

Table S1. Information of included GWAS data

Trait	GWAS ID/PMID	case/control	SNPs Numbers
Neonatal jaundice from other and unspecified causes	finn-b-P16_NEONTAL_JAUND_OTH_UNSP_CAUSES	133/218,608	16,380,466
Age at menarche	28436984	182 416	NA
Age at first birth	ebi-a-GCST90000048	418,758	10,766,720
Age at first sexual intercourse	ebi-a-GCST90000047	397,338	16,359,424
Illnesses of the mother: high blood pressure	PheCode : 642.1	NA	NA
Diabetes or abnormal glucose tolerance complicating pregnancy	GCST90044484	113/247427	NA
Maternal smoking around birth	GCST90041001-GCST90042000/GCST90041844/	391992	NA

Table S2. The information of included IVs for exposure.

Table S3. The information of Proxy SNPs records

Primary SNPs	Proxy SNPs
rs112282597	rs1864885
rs359240	rs359243
rs72704712	rs12588538
rs6744794	rs2341463
rs12714592	rs34007974
rs112523595	rs34495106
rs186723454	rs115882849
rs4728298	rs11772444
rs9643087	rs1039137
rs112880127	rs11198323
rs9581878	rs9581877
rs9536994	rs7984067
rs34155040	rs34939493
rs141164370	rs11587537
rs2461794	rs67451924
rs3111740	rs2952857
rs7465046	rs4464946
rs117008125	rs77796009
rs7256078	rs7254989
rs62229409	rs138792816
rs2028652	rs2920464
rs664172	rs667282
rs9668905	rs10842232

rs12920941	rs12599643

Table S4 The results of MR-PRESSO Test

Outcome	Exposure	Raw		Outlier corrected		Global *p*	No. of outliers	Distortion *p*
		ORCI	*P*	ORCI	*P*			
Neonatal jaundice	Age at first birth	0.8725 (0.6197 - 1.2285)	0.4376	NA (NA - NA)	NA	0.109	NA	NA
	Age at first sexual intercourse	1.4587 (0.6028 - 3.5299)	0.4034	NA (NA - NA)	NA	0.142	NA	NA
	Age at menarche	0.921 (0.6602 - 1.2849)	0.6286	NA (NA - NA)	NA	0.195	NA	NA
	Diabetes or abnormal glucose tolerance complicating pregnancy	0.9843 (0.8987 - 1.078)	0.7466	NA (NA - NA)	NA	0.444	NA	NA
	Illnesses of the mother high blood pressure	1.0812 (0.918 - 1.2735)	0.3809	NA (NA - NA)	NA	0.12	NA	NA
	Maternal smoking around birth	8.5227 (2.2084 - 32.8914)	0.0111	NA (NA - NA)	NA	0.615	NA	NA
	Maternal smoking around birth(both)	1943.7047 (0.1696 - 22280478.3427)	0.1634	NA (NA - NA)	NA	0.267	NA	NA
	Maternal smoking around birth(female)	1.6148 (0.021 - 124.1448)	0.8302	NA (NA - NA)	NA	0.076	NA	NA

Table S5. Statistical power calculations for each exposure-outcome pair in the two-sample Mendelian randomization analysis.

Outcome	Exposure	IVW_OR	Exposure_R2	N	K	Power
NJFO AUC	Age at first birth	0.821954431	0.005684177	218741	0.000608025	5.27%
NJFO AUC	Maternal smoking around birth	8.522706798	0.001158507	218741	0.000608025	84.85%
NJFO AUC	Illnesses of the mother high blood pressure	1.081203071	0.000455818	218741	0.000608025	5.00%
NJFO AUC	Diabetes or abnormal glucose tolerance complicating pregnancy	0.984278545	0.000371074	218741	0.000608025	5.00%
NJFO AUC	Age at menarche	0.906005959	0.069211454	218741	0.000608025	7.28%
NJFO AUC	Age at first sexual intercourse	1.33229816	0.02020217	218741	0.000608025	8.46%

© 2026 Geng W. et al.
